# Efficacy of technology-based interventions in psychosis: a systematic review and network meta-analysis

**DOI:** 10.1017/S0033291722003610

**Published:** 2023-10

**Authors:** Carla Morales-Pillado, Belén Fernández-Castilla, Teresa Sánchez-Gutiérrez, Eduardo González-Fraile, Sara Barbeito, Ana Calvo

**Affiliations:** 1 Department of Personality, Assessment and Clinical Psychology, School of Psychology, Universidad Complutense de Madrid, Madrid, Spain; 2 Faculty of Health Science, Universidad Internacional de La Rioja (UNIR), Madrid, Spain; 3 Department of Methodology of Behavioral and Health Sciences, Universidad Nacional de Educación a Distancia, Madrid, Spain

**Keywords:** eHealth, internet, mHealth, online interventions, psychosis, smartphone

## Abstract

**Background:**

Technology-based interventions (TBIs) are a useful approach when attempting to provide therapy to more patients with psychosis.

**Methods:**

Randomized controlled trials of outcomes of TBIs *v.* face-to-face interventions in psychosis were identified in a systematic search conducted in PubMed/Ovid MEDLINE. Data were extracted independently by two researchers, and standardized mean changes were pooled using a three-level model and network meta-analysis.

**Results:**

Fifty-eight studies were included. TBIs complementing treatment as usual (TAU) were generally superior to face-to-face interventions (*g* = 0.16, *p* ≤ 0.0001) and to specific outcomes, namely, neurocognition (*g* = 0.13, *p* ≤ 0.0001), functioning (*g* = 0.25, *p* = 0.006), and social cognition (*g* = 0.32, *p* ≤ 0.05). Based on the network meta-analysis, the effect of two TBIs differed significantly from zero; these were the TBIs cognitive training for the neurocognitive outcome [*g* = 0.16; 95% confidence interval (CI) 0.09–0.23] and cognitive behavioral therapy for quality of life (*g* = 1.27; 95% CI 0.46–2.08). The variables educational level, type of medication, frequency of the intervention, and contact during the intervention moderated the effectiveness of TBIs over face-to-face interventions in neurocognition and symptomatology.

**Conclusions:**

TBIs are effective for the management of neurocognition, symptomatology, functioning, social cognition, and quality of life outcomes in patients with psychosis. The results of the network meta-analysis showed the efficacy of some TBIs for neurocognition, symptomatology, and quality of life. Therefore, TBIs should be considered a complement to TAU in patients with psychosis.

## Introduction

Technology-based interventions (TBIs), including computer- and Internet-based interventions, mobile interventions, health applications, social media interventions, and interventions based on technological devices, could be an effective, accessible, and inexpensive option for delivering evidence-based approaches to patients with psychosis (Berry, Lobban, Emsley, & Bucci, 2016; Firth et al., 2016).

Although the use of technological resources in mental health is recent (Singla et al., 2020; Torous, Chan, Yellowlees, & Boland, 2016), the COVID-19 pandemic has led to the recommendation of online mental health interventions to guarantee delivery of care and safety (Moreno et al., 2020). Such resources now play a key role in mental health management (Vieta, Pérez, & Arango, 2020), and their accessibility, use, and adaptation have been prioritized (Torous, Myrick, Rauseo-Ricupero, & Firth, 2020).

In the case of psychosis, systematic reviews have been published on the acceptability of online interventions (Berry et al., 2016) and their effectiveness with respect to social media interventions (Välimäki, Athanasopoulou, Lahti, & Adams, 2016), adherence to web-based and mobile technologies (Killikelly, He, Reeder, & Wykes, 2017), feasibility of smartphone and mobile technology for clinically high-risk and early psychosis patients (Camacho, Levin, & Torous, 2019), and use of technological devices (Bonet et al., 2017; Firth et al., 2016). Only two systematic reviews of TBIs in psychosis have been published (Alvarez-Jimenez et al., 2014; Van Der Krieke, Wunderink, Emerencia, De Jonge, & Sytema, 2014), and neither exclusively covers randomized controlled trials (RCTs). The first systematic review focuses on the acceptability, feasibility, and benefits of online and mobile-based interventions for patients with psychosis and concludes that TBIs are as effective as standard treatment. The second focuses on self-management through various technological resources compared with standard treatment and reaches similar conclusions about face-to-face interventions. However, no systematic review or meta-analysis has focused on the effectiveness of TBIs in patients with psychosis and only includes studies with well-designed RCTs. To the best of our knowledge, this is the first systematic review and meta-analysis to compare the efficacy of TBIs over standard face-to-face interventions in patients with psychotic disorders in RCTs.

The aims of this meta-analysis are as follows: (1) to quantitatively synthesize the results from studies that test whether the use of TBIs as a complement to treatment as usual (TAU) leads to the same results as conventional face-to face interventions in patients with psychosis for a series of outcomes, namely, neurocognition, symptoms (positive and negative), functioning, social cognition, and quality of life; (2) to analyze which study characteristics favor or hamper the effectiveness of TBIs (e.g. moderator analysis); and (3) to perform a network meta-analysis to explore which treatment leads to a greater improvement for each outcome.

## Methods

Following the indications of the Preferred Reporting Items for Systematic Reviews and Meta-analyses (PRISMA-P) guidelines (Moher et al., 2015), articles were systematically searched for in PubMed/Ovid and MEDLINE until September 2020. The detailed search syntax and search strings, which were based on the PICO format (Higgins et al., 2019), are presented in eAppendix 1 in the online Supplementary material.

### Selection criteria

Studies were included in the systematic review if they met the following criteria: (1) articles reporting on RCTs that compare the efficacy of TBI as a complement to TAU with face-to-face interventions; (2) participants were diagnosed with first-episode psychosis (FEP), high risk of psychosis, schizophrenia, schizoaffective or schizophreniform psychotic disorder, and non-affective psychosis; and (3) the article had been peer-reviewed and published, or was currently in press, in English.

Studies were excluded if participants were diagnosed with a psychotic disorder due to another medical condition or substance-induced psychosis, and if the neuropsychological outcomes only required magnetic resonance imaging-based assessment.

### Data extraction

Titles and abstracts were screened independently by two researchers (EG-F, CM-P) to determine which manuscripts proceeded to full-text review. In the case of uncertainty, the full-text manuscript was retrieved. The same two authors independently reviewed full-text manuscripts to establish which of those met the inclusion criteria. Disagreements were resolved through discussion. A third reviewer (AC) was assigned to resolve any disagreement.

### Risk of bias

The risk of bias of the RCTs was assessed using the Cochrane Collaboration's tool for assessing risk of bias in randomized trials (Higgins et al., 2011). Two independent researchers (CM-P and AC) carried out this assessment. Disagreements were resolved through discussion.

### Statistical analysis

The effect size calculated for each RCT was the standardized mean change (SMC). The SMC is calculated by subtracting the difference between the post- and the pre-measures of the control group (CG) from the difference between post- and pre-measures of the intervention group (IG) and dividing it by the pooled standard deviation (s.d.) of the difference. To calculate the variance of the SMC, it is necessary to know the correlation between the pre- and post-measures. However, since this information was never reported in primary studies, we imputed a moderate correlation of 0.30. A positive effect size indicates that the IG experienced a greater improvement than the CG. Given that the effect size SMC might be overestimated if the sample size is small, we converted SMC to Hedges *g* (Hedges, 1981) to obtain more precise SMCs.

In total, 618 SMCs were calculated within 44 different studies. Several effect sizes were detected within the studies because studies included multiple dependent variables (i.e. attention, memory, executive function) and/or used multiple instruments (i.e. PANSS, CGI, MATRICS, CPT, GAF). In order to model the statistical dependency that emerges when several SMCs are calculated for the same sample and/or for the same study, a three-level model was applied (Cheung, 2014; Van den Noortgate, López-López, Marín-Martínez, & Sánchez-Meca, 2013, 2015). This takes into account the fact that effect sizes (level 1) are nested within different outcomes (level 2) nested within studies (level 3). TBI groups were categorized according to the descriptions offered by the different studies and are presented in eAppendix 2 in the online Supplementary material.

The analyses were performed in the following order. First, a combined SMC was obtained for each clinical outcome separately, that is, a combined SMC was obtained for neurocognition, symptomatology (positive and negative), functioning, social functioning, and quality of life. Each of these overall SMCs represents how much more effective TBIs combined with TAU are than standard face-to-face interventions. TAU could be any standard intervention that patients were already participating in or receiving (e.g. pharmacotherapy). The TBI was a complement to this intervention, that is, the TBI was neither the first choice nor the only one. Second, within each domain, separate meta-analyses were performed for each type of TBI, with these analyses being, at the same time, disaggregated according to the type of CG used. To be able to analyze the type of CG, three categories were created, as follows: (1) CG Psychotherapy, (2) CG Technology, and (3) CG Pharmacotherapy-only. CG Psychotherapy refers to any CG that used psychological and psychoeducational techniques plus pharmacotherapy; CG Technology refers to any CG that used non-psychotherapeutic TBIs, such as computer games, computer tasks, or video and television programs, plus pharmacotherapy. Finally, CG Pharmacotherapy-only refers to any CG that used only drugs, with no other intervention. The third step of the analyses involved analyzing moderator variables for those TBIs in which there were more than 20 effect sizes available within each outcome.^†^^1^ Moderator analyses were carried out by entering fixed moderator variables (representing the characteristics of the studies) into the three-level models. The variable ‘type of control group’ was also included in all meta-regressions to control for possible differences in the moderator variables across these CGs. The selected moderator variables included were related to sociodemographic variables and clinical interventions (Caron, Lecomte, Stip, & Renaud, 2005; Fiszdon, Kurtz, Parente, & Choi, 2020; Turner et al., 2020), as follows: educational level (measured according to the years of education specified in each RCT, generally from the age of initiation of compulsory schooling), type of medication (atypical antipsychotic, typical antipsychotic, antipsychotic without differentiation, only antipsychotics), dose of medication (measured as milligrams of drug), duration of intervention (measured in weeks), frequency of the intervention (measured in days of session per 15 days), type of TBI [computerized, interventions that use computers, the Internet, platforms and computer programs, or online (using mobile applications)], sex, age, type of contact during the intervention (with therapists, with other patients, with both groups, or no contact), and setting (clinic, home, or both). Fourth, a network meta-analysis was performed to investigate which treatment led to a greater improvement for each domain. With this methodology, direct and indirect estimates of the effectiveness of each online intervention are pooled to obtain more precise estimates (Salanti, 2012). The resulting rank of therapies (*p* scores, Rücker & Schwarzer, 2015) highlights the TBIs that are more effective for each type of outcome. In the network meta-analysis, it is important to comply with the transitivity assumption, which states that studies have to be homogeneous in terms of their characteristics so that differences observed across types of interventions are not due to the differential distribution of effect modifiers across studies (Jansen & Naci, 2013). Therefore, homogeneous groups of studies were created based on two relevant variables: diagnosis and average age of the participants. On the one hand, studies were divided based on the mean age of the participants, that is, mean age above 25 and below 25. This cut-off is based on previous studies with a similar sample (Bucci et al., 2018a, 2018b; Holzer et al., 2014; Østergaard Christensen et al., 2014). Two separate network meta-analyses were applied on each subset of studies. As the mean age of the participants was below 25 in very few studies, only the subset of studies with a mean age over 25 was analyzed. Studies were further divided based on diagnosis, as follows: schizophrenia (i.e. this category encompassed studies that labeled their patients as schizophrenia, schizophrenia/another psychotic disorder, schizophrenia/psychosis, schizophrenia/schizoaffective, schizophrenia/schizoaffective/psychosis, schizophrenia/schizophreniform/schizoaffective and non-affective psychosis) and FEP (i.e. studies that labeled patients as FEP, psychotic disorder, and one that included early psychosis, and psychosis/high risk of psychosis). In each of the studies included, at least 60% of the sample met the criteria for psychosis. Inconsistency between direct and indirect effects was evaluated through the node-splitting test (Dias, Welton, Caldwell, & Ades, 2010). Finally, in order to evaluate the possible presence of publication bias, funnel plots were created for each domain, and three-level Egger regression tests were carried out (Fernández-Castilla et al., 2021).

## Results

The flow diagram of the study selection process is illustrated in eAppendix 3, online Supplementary material, and the characteristics of all the studies included are described in online Supplementary Table S1

The meta-analysis included 58 studies, Bell, Fiszdon, Greig, Wexler, and Bryson (2007), Bell, Zito, Greig, and Wexler (2008), Bellucci, Glaberman, and Haslam (2003), Bryce et al. (2018), Cavallaro et al. (2009), Choi et al. (2018), Contreras, Tan, Lee, Castle, and Rossell (2018), d'Amato et al. (2011), Eack et al. (2009), Fisher, Holland, Merzenich, and Vinogradov (2009), Fisher, Holland, Merzenich, and Vinogradov (2010), Fisher, Mellon, Wolkowitz, and Vinogradov (2016), Fisher et al. (2015), Fiszdon, Kurtz, Choi, Bell, and Martino (2016), Garety et al. (2015), Garrido et al. (2013), Gottlieb et al. (2017), Greig, Zito, Wexler, Fiszdon, and Bell (2007), Hogarty et al. (2004), Hooker et al. (2012), Iwata et al. (2017), Jahshan, Vinogradov, Wynn, Hellemann, and Green (2019), Krzystanek, Borkowski, Skałacka, and Krysta (2019), Kukla, Bell, and Lysaker (2018), Kurtz, Mueser, Thime, Corbera, and Wexler (2015), Kurtz, Seltzer, Shagan, Thime, and Wexler (2007), Lado-Codesido, Méndez Pérez, Mateos, Olivares, and García Caballero (2019), Lee (2013), Leff, Williams, Huckvale, Arbuthnot, and Leff (2013), Lindenmayer et al. (2018), Mahncke et al. (2019), Maroño Souto et al. (2018), Matsuda et al. (2018), Miley et al. (2020), Moritz et al. (2011, 2015a, 2015b, 2016), Pitkänen et al. (2012), Popov et al. (2011, 2014), Rass et al. (2012), Rodewald et al. (2011), Sachs et al. (2012), Schlosser et al. (2018), Shuib, Ahmad, Osman, and Alwi (2019), Tessier et al. (2020), Urben, Pihet, Jaugey, Halfon, and Holzer (2012), Van den Noortgate, López-López, Marín-Martínez, and Sánchez-Meca (2013), Vauth et al. (2005), Vinogradov et al. (2009), Vreeland et al. (2006), Wölwer et al. (2005), Xu et al. (2019), and Zhu et al. (2020) (online Supplementary Table S1) reporting 618 effect sizes or SMCs. The total sample comprised of 4394 participants, of whom 64.31% were male. The mean age was 36.58 (s.d. = 9.50) years, and the mean educational level was 11.37 (s.d. = 2.97) years. The average duration of the interventions was 12 weeks, mostly without contact with other patients or therapists (30 CGs and 25 IGs) and with prescription of antipsychotics (17 studies did not report this). In 40% of the studies, patients had at least three sessions a week, whereas in the remaining 60% of the studies, patients had fewer than three sessions a week. In most of the studies, the intervention occurred in a clinic (81%), in nine studies the intervention occurred at home, and in two studies the intervention occurred both in a clinic and at home.

Forty-nine interventions were computerized or based on other devices, and nine were online, using mobile applications and health applications. Forty-three studies showed significant improvements compared with the CG, and eight showed no differences between the groups. Thirty-four studies measured neurocognition, 29 measured symptomatology (19 positive symptomatology and 18 negative symptomatology), 23 measured functioning, 17 measured social cognition, and 15 measured quality of life.

Cognitive training therapy was a common component of most TBIs (26 studies alone and 15 studies with another TBI therapy), compared with face-to-face group psychotherapy interventions (31 studies).

### Overall analyses

The overall SMC showed that, globally, TBIs were slightly superior to face-to-face interventions, as a complement to TAU (*g* = 0.16, s.e. = 0.03, *z* = 5.36, *p* ≤ 0.0001, between-studies variance = 0.01, within-study variance = 0.17), although the effect is small. The overall SMC for each outcome can be found in Table 1.

**Table 1.** Comparison of TBIs as a complement to TAU *v.* face-to-face interventions, separated for outcomes

	*k*	*d*	s.e.	*z*	*p* value	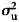	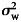
Neurocognition							
Total_TBI	321	0.133	0.032	4.135	<0.0001	0.016 (34)	0.035 (321)
Symptomatology							
Total_TBI	117	0.114	0.069	1.642	0.101	0.006 (29)	0.377 (117)
Functioning							
Total_TBI	98	0.245	0.090	2.724	0.006	0.057 (23)	0.274 (98)
Social cognition							
Total_TBI	44	0.317	0.129	2.459	<0.05	0.000 (17)	0.596 (44)
Quality of life							
Total_TBI	38	0.142	0.080	1.778	0.076	0.046 (16)	0.000 (38)

*k*, number of effect sizes within each category; *d*, overall effect size; s.e., standard error; 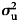, between-studies variance. In this column, the number of studies analyzed is indicated in brackets; 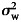, between-outcomes variance. In this column, the number of effect sizes analyzed is indicated in brackets.

#### Neurocognitive outcomes

A significant effect for the outcome neurocognition was observed in the analyses of TBIs compared with all CGs together in 34 studies (*g* = 0.13, s.e. = 0.03, *z* = 4.14, *p* ≤ 0.0001). Specifically, significant effects were observed in the analyses of TBI Cognitive training compared with the CG Psychotherapy (*g* = 0.29, s.e. = 0.09, *z* = 3.11, *p* ≤ 0.01) and with CG Technology (*g* = 0.19, s.e. = 0.06, *z* = 3.33, *p* ≤ ≤0.001). Also, the combination of TBI Cognitive Training, cognitive behavioral therapy (CBT) and vocational therapy led to a significant SMC compared with a CG Psychotherapy (*g* = 0.11, s.e. = 0.05, *z* = 2.01, *p* < 0.05). On the other hand, TBI CBT worked worse than the CG Psychotherapy (*g* = −0.55, s.e. = 0.14, *z* = −3.86, *p* < 0.001). The results of the remaining TBIs available for this outcome are given in eTable 1 in eAppendix 4.

Based on the network meta-analysis, the TBI combining Cognitive training with Vocational therapy was the one that worked best for the neurocognitive outcome (*p* = 0.94), followed by TBI Cognitive training (*p* = 0.87). However, the network meta-analysis results showed that the overall effectiveness of TBI Cognitive training was statistically different from zero, whereas the 95% confidence interval (CI) of the overall effect estimated for TBIs combining Cognitive training with Vocational therapy included the value of zero (eTable 8 in eAppendix 5). These results were also found for the subset of studies where the mean age of the participants was above 25 (eTable 9 in eAppendix 5), and for the subset of studies where participants were diagnosed with schizophrenia (eTable 11 in eAppendix 5). However, for the group of patients with FEP, the best intervention was CG Technology, although this effect was not statistically different from zero. Even so, these results must be interpreted with caution, because inconsistency was found between the direct and indirect effects of the comparison between TBI Cognitive training and CG Psychotherapy. More details about the results of the network meta-analysis can found in eAppendix 5. Finally, no evidence of publication bias was detected in either the funnel plot (eFig. 31) or in the three-level Egger regression test (eTable 34).

*Moderators*: There was an effect of educational level for TBI Cognitive training in the IG. On average, across types of CGs, the higher the educational level of the IG, the greater the difference between the CG and the IG (*b* = 0.34, s.e. = 0.02, *p* < 0.05).

There was also an effect of the frequency of the sessions for the TBI combining Cognitive training and Social cognition: the more frequent the sessions were, the smaller the difference observed between the recovery of the IG and the CG (*b* = −0.05, s.e. = 0.02, *p* < 0.05). No effect of the moderator variables was found for the remaining interventions. More results can be found in eTables 26–28 from eAppendix 6.

#### Symptomatology outcomes

No significant effect was observed when TBIs were compared with all CGs together in this domain (*g* = 0.11, s.e. = 0.07, *z* = 1.64, *p* = 0.101). However, TBI Cognitive training performed significantly better than CG Psychotherapy (*g* = 0.23, s.e. = 0.10, *z* = 2.45, *p* < 0.05) and TBI CBT also performed better than CG Psychotherapy (*g* = 0.63, s.e. = 0.20, *z* = 3.18, *p* < 0.01). On the contrary, TBI Psychoeducation led to worse results than CG Psychotherapy (*g* = −0.37, s.e. = 0.13, *z* = −2.94, *p* < 0.01). The results of the other TBIs available for this outcome can be found in eTable 2 in eAppendix 4.

Based on the results of the network meta-analysis, the TBI CBT was the one that worked best for the symptomatology outcome (*p* = 0.84). This was also true for the sample of patients with FEP (*p* = 0.99), although the overall effect size was only significantly different from zero in the subset of studies where participants were diagnosed with FEP (eTable 14). In the sample of patients with schizophrenia and in the sample of patients with psychotic disorders aged over 25 years, the best intervention was TBI Cognitive training combined with TBI Social cognition, although, similarly, these overall effect sizes did not differ significantly from zero. Inconsistent effect sizes were found when all studies were analyzed together, although these inconsistencies disappeared in the subgroup analyses. More details about the results of the network meta-analysis can be found in eAppendix 5. Finally, no evidence of publication bias was detected in either the funnel plot (eFig. 32) or the three-level Egger regression test (eTable 34).

**Fig. 1.**
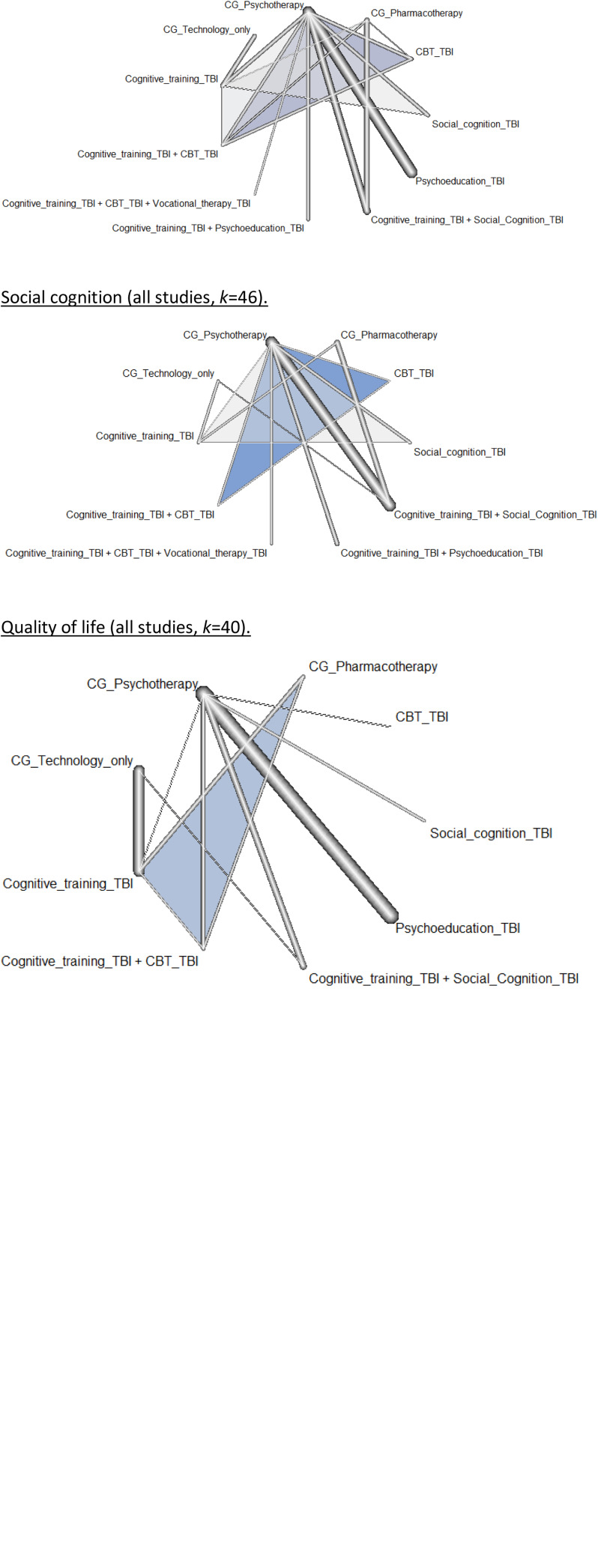
Network plot for the interventions for the different outcomes. Neurocognition (all studies, *k* *=* 351); symptomatology (all studies, *k* = 130); functioning (all studies, *k* = 130); social cognition (all studies, *k* = 46); quality of life (all studies, *k* = 40). The thickness of the lines is proportional to the number of studies that reported the comparison between those treatments. CG, control group.

Subanalyses were carried out based only on effects that referred to positive symptomatology (*k* = 31) and effects that referred to negative symptomatology (*k* = 23). For both outcomes, the overall effect was non-significant and very close to zero (positive symptomatology, *g* = −0.05; and negative symptomatology, *g* = 0.08). No therapy (or combination of therapies) showed an effect for positive symptomatology. For negative symptomatology, CBT_TBI showed effectiveness over CG Psychotherapy (*g* = 0.91, s.e. = 0.45, *p* < 0.05), although this result is only based on one effect size. Furthermore, TBI Psychoeducation performed worse than CG Psychotherapy (*g* = −0.56, s.e. = 0.25, *p* < 0.05), although once again this result has to be interpreted with caution because it is only based on two effect sizes. Extended results from these outcomes can be found in eTables 3 and 4 in eAppendix 4. No moderator analyses were carried out in these subgroups because there were fewer than 20 effect sizes for each treatment (or combination of treatments) (Fig. 1).

*Moderators*: An effect of the variable ‘Contact during intervention in the CG’ was observed in the cognitive training TBIs. Controlling by type of CGs, when the contact is with ‘Therapist’ instead of with ‘Others’, the difference between the CG and the IG increases (*b* = 0.51, s.e. = 0.24, *p* ≤ 0.05).

Furthermore, there was an effect of the type of medication. For TBI Cognitive training, the overall effect in studies where participants used an atypical antipsychotic was on average larger than the overall effect in studies where participants used a typical antipsychotic (*b* = 0.38, s.e. = 0.18, *p* ≤ 0.05). More results can be found in eAppendix 6.

#### Functioning

A significant overall effect was observed for TBIs compared with all CGs together (*g* = 0.25, s.e. = 0.09, *z* = 2.72, *p* ≤ 0.01). The TBI Social Cognition showed better results than GC Psychotherapy (*g* = 0.55, s.e. = 0.12, *z* = 4.67, *p* < 0.001), as did the combination of TBI Cognitive training and TBI Social Cognition (*g* = 0.65, s.e. = 0.26, *z* = 2.53, *p* < 0.05). In addition, TBI Psychoeducation also showed a marked effect compared with GC Psychotherapy (*g* = 0.31, s.e. = 0.074, *z* = 4.19, *p* < 0.001), as did the combination of TBIs CBT and Cognitive training compared with GC Psychotherapy (*g* = 0.32, s.e. = 0.13, *z* = 2.38, *p* < 0.05). The other overall effects of each TBI and their combination disaggregated by the type of CG can be found in eTable 5 in eAppendix 4.

Network meta-analysis results indicated that the combination of TBI Cognitive training and TBI Social cognition works best for this outcome (*p* = 0.95, see eTable 16). This finding was also true for the sample of patients with schizophrenia (*p* *=* 0.95, eTable 17) and for the sample of patients aged over 25 years (*p* = 0.92, see eTable 19), but not for the sample of patients with FEP, in which the best intervention was TBI Cognitive training combined with TBI Psychoeducation. Even so, none of the overall effects of these treatments were statistically different from zero compared with CG Technology. Extended results from these outcomes can be found in eAppendix 5.

No effect of the moderator variables was found for this outcome. Regarding the publication bias analyses, the funnel plot (eFig. 33) showed that two small studies reported very large, positive effects, whereas no small study reported a very large, negative effect; therefore, publication bias could exist. Given that the three-level Egger regression test confirmed this result (eTable 34), results for functioning (overall effect = 0.25) might be slightly inflated.

#### Social cognition

A significant overall effect was observed for TBIs compared with all CGs together (*g* = 0.32, s.e. = 0.13, *z* = 2.46, *p* ≤ 0.05). However, when each type of TBI was studied separately, the only significant effect observed was for the comparison between TBI Cognitive training and CG Technology (*g* = 0.60, s.e. = 0.20, *z* = 2.95, *p* < 0.01). The other overall effects disaggregated by the type of CG can be found in eTable 6 in eAppendix 4.

Network meta-analysis results indicated that TBI Cognitive training combined with the TBI Social cognition works best for this outcome (*p* = 0.70, see eTable 20). This finding was also true for the studies in which the mean age is over 25 (*p* = 0.72, see eTable 21) and for the sample of patients with schizophrenia (*p* = 0.72, see eTable 22). However, none of these overall effect sizes were statistically different from zero. There were no effect sizes available for participants who had experienced FEP. More information is provided in eAppendix 5.

No moderator variables proved to be statistically significant. Finally, no evidence of publication bias was detected in either the funnel plot or in the three-level Egger regression test for this domain.

#### Quality of life

The overall SMC for this outcome was not significant (*g* = 0.14, s.e. = 0.08, *z* = 1.78, *p* = 0.076), meaning that TBIs did not perform better than the CGs. When analyses were performed separately for each TBI, the only effect found was for TBI CBT (*g* = 1.24, s.e. = 0.48, *z* = 2.59, *p* ≤ 0.01), although this result is only based on one effect size. The other overall effects disaggregated by the type of CG can be seen in eTable 7 from eAppendix 4.

The results from the network meta-analysis showed that TBI CBT was statistically better than GC Technology in the general analysis (*p* = 0.99, see eTable 23). However, the node-splitting method detected inconsistent effects, specifically between the direct and indirect evidence of the effectiveness of Cognitive Training over CG Psychotherapy. When only studies with a sample with a mean age over 25 were analyzed, these inconsistencies disappeared, although the best treatment in the ranking became Social cognition_TBI (*p* = 0.82, see eTable 24). For the subanalyses of studies with a sample of participants with schizophrenia, the best treatment was also Social cognition_TBI (*p* = 0.84, see eTable 25), although, once again, inconsistent effects were found. Therefore, results from that domain should be interpreted with caution. More details about the results of the network meta-analysis can be found in eAppendix 5.

## Discussion

The present study demonstrated that TBIs, as a complement to TAU, are effective compared with face-to-face interventions for patients with psychosis. Based on these findings, patients who received TBIs subsequently performed better on average in various outcomes, namely, neurocognition, functioning, and social cognition.

Therefore, TBIs should be considered a complement to TAU and face-to-face interventions, because they were at least as effective as first-line interventions (Van Der Krieke et al., 2014). Furthermore, TBIs have the added value of being able to reach more people, especially those affected by problems of accessibility, mobility, and stigmatization (Sánchez-Gutiérrez, Barbeito, & Calvo, 2017; Wallin, Mattsson, & Olsson, 2016).

The findings of this study highlight the effectiveness of TBIs, which could become a standardized complement to therapy and, therefore, an effective formula for maintaining the long-term effectiveness of face-to-face interventions that have already demonstrated their short- and medium-term efficacy. Numerous specific face-to-face psychotherapeutic programs developed to address FEP (Bertelsen et al., 2008; Calvo et al., 2014; Craig et al., 2004; Gleeson et al., 2013) showed that after completion, their benefits were not sustained in the long term (Bertelsen et al., 2008; Calvo et al., 2015; Gafoor et al., 2010; Gleeson et al., 2013; Secher et al., 2015). Still, more research is needed on TBIs, as they could help to maintain long-term effectiveness.

Our results could represent a major advance when offering treatment to patients with psychosis, since the resources are more accessible, flexible, and cost-effective than face-to-face therapies.

Implementation of TBIs as part of a mental health project requires an evidence-based digital inclusion strategy that covers education and training in basic skills for using technological devices. This training would help digitally excluded populations to access these services (Robotham, Satkunanathan, Doughty, & Wykes, 2016). Good management and use of technology is generally assumed (Bucci et al., 2018a, 2018b; Firth et al., 2016), although some studies take into account the possibility that perhaps not all people with psychosis are represented (Gay, Torous, Joseph, Pandya, & Duckworth, 2016).

### Neurocognition

TBI Cognitive training with TBI Vocational therapy added to TAU showed better results than other face-to-face interventions (Pharmacotherapy, Psychotherapy, or Technology). Our results show that TBI Cognitive training added to TAU (alone or in combination with other therapies) was effective in improving neurocognition. While significant effects of certain therapies were found in the main analyses, the network meta-analysis only revealed evidence that TBI Cognitive training is significantly better than Technology, both in the general analysis and for the group aged above 25 years with schizophrenia. This is because more studies reported comparisons between these interventions (TBI Cognitive training *v.* Technology), thus ensuring sufficient statistical power to detect an effect. However, given that other interventions were less studied, fewer comparisons are available. Since this directly affects statistical power, the probability of detecting an existing effect is much lower. Moreover, cognitive training has a long-studied impact on cognition (Kurtz, Moberg, Gur, & Gur, 2001; McGurk, Twamley, Sitzer, McHugo, & Mueser, 2007). Implementing TBIs yields the same results as conventional interventions in terms of the convenience of cognitive training for cognitive impairments in psychosis, because only the way exercises are administered varies, and not their content. Our focus is limited to the variable neurocognition. However, it would be very interesting to evaluate which TBIs would be better for implementing all the outcomes at the same time and whether, in this case, TBI cognitive training could be superior to the others.

Similarly, it was not surprising that other TBIs such as CBT and Social cognition were not as effective as Pharmacotherapy or face-to-face interventions in neurocognition, given that they do not focus on improvements in this area (Lally & MacCabe, 2015; McCleery & Nuechterlein, 2019).

### Symptomatology

TBIs were also effective in terms of symptomatology. In this case, TBI CBT added to TAU was more effective than Pharmacotherapy alone, which is usually the first-line treatment for psychosis (Haddad & Correll, 2018; Leucht et al., 2017). The network meta-analysis revealed similar findings, namely, TBI CBT combined with TAU was significantly better than Technology in the FEP group. Therefore, TBI CBT added to TAU is an efficacious intervention for improving the symptomatology of affected patients and could act as a complement to pharmacotherapy. In addition, the implementation of mobile applications and internet platforms that offer this type of treatment is recommended (Mehl, Werner, & Lincoln, 2015). Cognitive training interventions have also proven to be very effective, probably owing to the large number of studies for this type of intervention.

### Functioning, social cognition, and quality of life

Large overall SMCs were observed for functioning, social cognition, and quality of life, although these results are based on a smaller number of studies (98, 44, and 38, respectively). Even so, for functioning and quality of life, Pharmacotherapy is the third most effective therapy, and the second most effective for social cognition (eAppendix 5). A similar result was not found for neurocognition or symptomatology. This is interesting, because medication has been generally associated with an improvement in neurocognition and symptoms and not so much with an improvement in functioning, social cognition, and quality of life (Bobes, Garcia-Portilla, Bascaran, Saiz, & Bouzoño, 2007; Kucharska-Pietura & Mortimer, 2013; Lally & MacCabe, 2015).

However, the interventions grouped under the heading of Psychotherapy were the least effective, again, probably owing to the number of studies that focus on cognitive training, compared with the remaining interventions. Even so, results for TBIs are very positive in these outcomes.

Therefore, TBIs are a viable strategy for patients with psychosis. Cognitive training plus Social cognition is the best option for improving all outcomes, together with Pharmacotherapy in the case of face-to-face interventions. Furthermore, a previous study proposed that combining cognitive training and social cognition could be an effective approach in the treatment of psychosis (Lindenmayer et al., 2013).

### Moderator variables

Study characteristics have few repercussions for the observed effect sizes. The only aspects worthy of note are in neurocognition and symptomatology outcomes, where the study characteristics that did influence the effect sizes observed were educational level, frequency of the intervention, type of medication, and whether there was contact, during the intervention, with therapists, with others, with both, or with no one. Patients with a higher educational level also have better academic results and have spent longer in education. Therefore, highly educated patients could be more familiar with learning processes. The TBI Cognitive training intervention consists partly of cognitive tasks to improve attention, working memory, verbal and non-verbal episodic memory, executive function, language processing, exercises, and repeated practices (Biagianti, Castellaro, & Brambilla, 2021). This could explain why educational level was a significant moderator.

Results for the variable ‘frequency of the intervention’ show that having very frequent and continuous sessions is not related to better results. Future studies need to identify the appropriate number and frequency of the sessions that lead to better results, as well as the number of sessions at which improvements are no longer observed.

Although investigation of the intervention and patient factors associated with the effects of TBI is relevant, no hypotheses propose variables that potentially influence the effects of the treatment administered (Grant, Lawrence, Preti, Wykes, & Cella, 2017). Some studies reported moderators for specific variables, such as adherence. The duration of the intervention can improve adherence, in contrast with male sex and younger age, which impair it (Killikelly et al., 2017).

We did not obtain significant results in these outcomes, possibly due to the lack of statistical power. Furthermore, given that we only carried out moderator analyses in interventions that had at least 20 effect sizes, we are unable to hypothesize about the effect of the moderators for interventions that had a smaller number of effect sizes.

### Limitations

The findings reported here are subject to a series of limitations. First, CGs might differ across the studies. The present study tried to group CGs into three categories (i.e. Psychotherapy, Technology, and Pharmacotherapy-only), although a more detailed description of the control condition was sometimes lacking in primary studies. Moreover, in some studies, face-to-face intervention was compared with the same intervention after adding TBI, meaning that the technology-based approach acted as a supplement to standard care. Second, for some domains (i.e. social cognition and quality of life), the number of effect sizes was small, and there was insufficient power to detect the effect of moderator variables. In addition, inconsistent effects were found in the network meta-analysis, although most were resolved when subgroups of studies were created based on the mean age of the participants and their diagnosis.

## Conclusions

The findings of this systematic review and meta-analysis demonstrated that TBIs were an effective option for the management of neurocognition, symptomatology, functioning, social cognition, and quality of life in patients with psychosis. Therefore, TBIs can complement TAU and face-to-face interventions.

TBIs could foster recovery in patients with psychosis beyond the capacity of face-to-face interventions and mainly when environmental circumstances limit access to on-site therapy. TBI will enable patients and professionals to develop flexible and personalized interventions to ensure patients' needs in such a way that therapeutic objectives can be more personalized.

## Notes

1 We only selected TBIs for which at least 20 effect sizes were available to carry out the moderator analyses, thus ensuring sufficient power to detect an effect of the moderator.

## Footnotes

† The notes appear after the main text.

## Supplementary material

For supplementary material accompanying this paper visit https://doi.org/10.1017/S0033291722003610. click here to view supplementary material
