# Supplementary material for: Efficacy of technology-based interventions in psychosis: a systematic review and network meta-analysis
Source: Psychol Med. 2022 Dec 6;53(13):6304–15. doi: 10.1017/S0033291722003610 (PMC10520607; doi:10.1017/S0033291722003610)
Supplement: Supplementary file 1 [file S0033291722003610sup.zip › S0033291722003610sup001.doc]

**SUPPLEMENTARY MATERIAL**

**eAppendix 1. Search strategy**

The research question was framed based on the PICO format (P: participants; I: interventions: C: comparisons; O: outcomes), which is endorsed by the Cochrane Collaboration^1^.

- P: Participants: patients with severe mental disorder, that is, at least one disorder from First episode psychosis (FEP), high risk of psychosis, schizophrenia, schizoaffective, schizophreniform, psychotic disorder and non-affective psychosis.
- I: Types of studies: Randomized control trials to assess the efficacy of TBI, as a complement to TAU.
- C: Comparisons: the quantitative studies included had an intervention and a control group (patients with the same characteristics who did not receive the study treatment).
- O: Outcomes: neurocognition, symptomatology, functioning, social cognition, and quality of life.

The following search terms were used:

#1.	exp SCHIZOPHRENIA/

#2.	schizo$.mp.

#3.	psychotic$.mp.

#4.	psychosis.mp.

#5.	psychoses.mp.

#6.	FEP.ti,ab.

#7.	EOP.ti,ab.

#8.	Ultra high risk

#9.	Clinical high risk

#10.	Or/1-9

#11.	On-line.ti,ab,kw.

#12.	Online.ti,ab,kw.

#13.	internet.ti,ab,kw.

#14.	web.ti,ab,kw.

#15.	mobile.ti,ab,kw.

#16.	Smartphone.ti,ab,kw.

#17.	app.ti,ab,kw.

#18.	Computer.ti,ab,kw.

#19.	chatbot

#20.	Or /11-19

#21.	exp clinical trial/

#22.	exp randomized controlled trials/

#23.	exp double-blind method/

#24.	exp single-blind method/

#25.	exp cross-over studies/

#26.	randomized controlled trial.pt.

#27.	clinical trial.pt.

#28.	controlled clinical trial.pt.

#29.	(clinic$ adj2 trial).mp.

#30.	(random$ adj5 control$ adj5 trial$).mp.

#31.	(crossover or cross-over).mp.

#32.	((singl$ or double$ or trebl$ or tripl$) adj (blind$ or mask$)).mp.

#33.	randomi$.mp.

#34.	(random$ adj5 (assign$ or allocat$ or assort$ or reciev$)).mp.

#35.	or/21-34

#36.	10 AND 20 AND 35

The abstract, title and keyword(s) fields were searched in the database.

**eAppendix 2. Classification of TBIs groups**

Cognitive training_ TBI

Cognitive training_TBI + Vocational therapy_TBI

Cognitive training_TBI + Social cognition_TBI

Cognitive training_TBI + Psychoeducation_TBI

Cognitive training_TBI + CBT_TBI

Cognitive training_TBI + CBT_TBI + Vocational therapy_TBI

Social cognition_TBI

CBT_TBI

CBT_TBI + Vocational therapy_TBI

CBT_TBI + Mindfulness

Psychoeducation_TBI

Mindfulness_TBI

**eAppendix 3. Flow Diagram of Study Selection Process**

**eFigure 1. Flow chart representing systematic search and extraction procedure.**

**eAppendix 4. Overall effect for each outcome and disaggregated by the type of control group**

These tables contain the overall effect of each treatment compared to all control groups together (lines in bold) and then the overall effect of each treatment disaggregated by type of control groups. Sometimes the three-level model did not converge (due to a small number of effect sizes). In those cases, a standard random-effects model was fitted, and the cell in the column of the between-outcomes variance was left empty.

| **eTable 1. Neurocognition results. Meta-analytic estimates for each therapy (or combination of therapies) and for each type of control group.** | | | | | | | |
| --- | --- | --- | --- | --- | --- | --- | --- |
| **NEUROCOGNITION** | | | | | | | |
| **Types of intervention** | *k* | *g* | SE | Z | *p*-value |  |  |
| **Total_TBI** | 321 | 0.133 | 0.032 | 4.135 | <.0001 | 0.016  (34) | 0.035  (321) |
|  |  |  |  |  |  |  |  |
| *One therapy* |  |  |  |  |  |  |  |
| **CBT_TBI** |  |  |  |  |  |  |  |
| Psychotherapy | 6 | -0.547 | 0.142 | -3.860 | <.0001 | 0.000 (1) |  |
| **Cognitive training_TBI** | **184** | **0.202** | **0.047** | **4.221** | **<.0001** | **0.023 (21)** | **0.034**  **(184)** |
| Pharmacotherapy | 40 | 0.087 | 0.097 | 0.904 | .366 |  |  |
| Technology | 105 | 0.191 | 0.057 | 3.333 | <.001 |  |  |
| Psychotherapy | 39 | 0.290 | 0.093 | 3.107 | <.01 |  |  |
| **Social cognition_TBI** |  |  |  |  |  |  |  |
| Psychotherapy | 12 | -0.015 | 0.116 | -0.131 | .896 | 0.000 (2) |  |
| *Two therapies* |  |  |  |  |  |  |  |
| ***CBT_TBI +  Vocational therapy_TBI*** |  |  |  |  |  |  |  |
| Psychotherapy | 14 | -0.079 | 0.114 | -0.690 | .490 | 0.009 (2) | 0.000 (14) |
| ***CBT_TBI +  Cognitive training_TBI*** | **13** | **0.055** | **0.167** | **0.332** | **.740** | **0.000 (3)** | **0.227 (13)** |
| Pharmacotherapy | 3 | 0.017 | 0.392 | 0.043 | .966 |  |  |
| Technology | - | - | - | - | - |  |  |
| Psychotherapy | 10 | 0.062 | 0.199 | 0.310 | .756 |  |  |
| ***Cognitive training_TBI + Psychoeducation_TBI*** |  |  |  |  |  |  |  |
| Psychotherapy | 10 | 0.109 | 0.076 | 1.428 | .153 | 0.008 (1) |  |
| ***Cognitive training_TBI + Social cognition_TBI*** | **46** | **0.110** | **0.069** | **1.591** | **.112** | **0.003 (5)** | **0.102 (46)** |
| Pharmacotherapy | 11 | 0.151 | 0.175 | 0.862 | .389 |  |  |
| Technology | 1 | 0.270 | 0.629 | 0.429 | .668 |  |  |
| Psychotherapy | 34 | 0.073 | 0.111 | 0.653 | .514 |  |  |
| ***Cognitive training_TBI +  TAU*** |  |  |  |  |  |  |  |
| Pharmacotherapy | 12 | 0.310 | 0.224 | 1.383 | .167 | 0.000 (1) | 0.512 (12) |
| **Cognitive training_TBI + Vocational therapy_TBI** |  |  |  |  |  |  |  |
| Psychotherapy | 4 | 0.269 | 0.150 | 1.799 | .072 | 0.028 (1) |  |
| *Three therapies* |  |  |  |  |  |  |  |
| **Cognitive training_TBI + CBT_TBI +  Vocational therapy_TBI** |  |  |  |  |  |  |  |
| Psychotherapy | 32 | 0.109 | 0.054 | 2.005 | .045 | 0.000 (3) | 0.000 (32) |
| *Notes*. k= number of effect sizes within each category; *g* = overall effect size; SE= standard error; = between-studies variance. In this column, the number of studies analyzed is indicated in brackets; = between-outcomes variance. In this column, the number of effect sizes analyzed is indicated in brackets. | | | | | | | |

**eTable 2. Symptomatology results. Meta-analytic estimates for each therapy (or combination of therapies) and for each type of control group.**

| **SYMPTOMATOLOGY** | | | | | | | |
| --- | --- | --- | --- | --- | --- | --- | --- |
| **Types of intervention** | *k* | *g* | SE | Z | *p*-value |  |  |
| **Total_TBI** | 117 | 0.114 | 0.069 | 1.642 | .101 | 0.006 (29) | 0.377 (117) |
|  |  |  |  |  |  |  |  |
| *One therapy* |  |  |  |  |  |  |  |
| **CBT_TBI** | **20** | **0.122** | **0.306** | **0.399** | **.690** | **0.225 (3)** | **0.000 (20)** |
| Pharmacotherapy | 10 | -0.095 | 0.184 | -0.516 | .606 | 0.018 | 0.000 |
| Psychotherapy | 10 | 0.632 | 0.199 | 3.179 | <.01 |  |  |
| **Cognitive** **training_TBI** | **35** | **0.084** | **0.079** | **1.060** | **.289** | **0.029 (14)** | **0.000 (35)** |
| Pharmacotherapy | 8 | 0.084 | 0.147 | 0.575 | .566 |  |  |
| Technology | 8 | -0.179 | 0.136 | -1.323 | .186 |  |  |
| Psychotherapy | 19 | 0.232 | 0.095 | 2.452 | .014 |  |  |
| **Psychoeducation_TBI** | **17** | **-0.192** | **0.128** | **-1.504** | **.133** | **0.031 (3)** | **0.000 (17)** |
| Pharmacotherapy | 5 | -0.216 | 0.243 | -0.887 | .375 |  |  |
| Technology | 3 | 0.012 | 0.094 | 0.132 | .895 |  |  |
| Psychotherapy | 9 | -0.367 | 0.125 | -2.943 | <.01 |  |  |
| **Social cognition_TBI** |  |  |  |  |  |  |  |
| Psychotherapy | 10 | 0.188 | 0.114 | 1.656 | .098 | 0.000 (4) |  |
| *Two therapies* |  |  |  |  |  |  |  |
| **CBT_TBI +  Vocational therapy_TBI** |  |  |  |  |  |  |  |
| Psychotherapy | 4 | -0.256 | 0.207 | -1.237 | .216 | 0.000 (1) |  |
| **CBT_TBI + Cognitive training_TBI** |  |  |  |  |  |  |  |
| Pharmacotherapy | 3 | -0.171 | 0.209 | -0.847 | .397 | 0.000 (1) |  |
| **Cognitive training_TBI + Psychoeducation_TBI** |  |  |  |  |  |  |  |
| Psychotherapy | 3 | 0.135 | 0.129 | 1.047 | .296 | 0.000 (1) |  |
| **Cognitive training_TBI +  Social cognition_TBI** | **20** | **0.307** | **0.331** | **0.929** | **.353** | **0.000 (4)** | **2.090 (20)** |
| Pharmacotherapy | 3 | 0.227 | 0.867 | 0.261 | .794 |  | 2.217 |
| Psychotherapy | 17 | 0.323 | 0.370 | 0.871 | .383 |  |  |
| *Three therapies* |  |  |  |  |  |  |  |
| **Cognitive training_TBI + CBT_TBI + Vocational therapy_TBI**  Psychotherapy | 5 | 0.137 | 0.171 | 0.803 | .422 | 0.000 (2) |  |

*Notes*. k= number of effect sizes within each category; *g* = overall effect size; SE= standard error;= between-studies variance. In this column, the number of studies analyzed is indicated in brackets;= between-outcomes variance. In this column, the number of effect sizes analyzed is indicated in brackets.

| **eTable 3. Positive symptomatology results. Meta-analytic estimates for each therapy (or combination of therapies) and for each type of control group.** | | | | | | | |
| --- | --- | --- | --- | --- | --- | --- | --- |
| **POSITIVE SYMPTOMATOLOGY** | | | | | | | |
| **Types of intervention** | *k* | *g* | SE | Z | *p*-value |  |  |
| **Total_TBI** | 31 | -0.052 | 0.081 | -0.643 | .520 | 0.043 (19) | 0.000 (31) |
|  |  |  |  |  |  |  |  |
| *One therapy* |  |  |  |  |  |  |  |
| **CBT_TBI** | **7** | **-0.029** | **0.169** | **-0.174** | **.862** | **0.000 (7)** |  |
| Pharmacotherapy | 4 | -0.079 | 0.223 | -0.353 | .724 |  |  |
| Psychotherapy | 3 | 0.037 | 0.258 | 0.142 | .887 |  |  |
| **Cognitive** **training_TBI** | **9** | **0.119** | **0.117** | **1.011** | **.312** | **0.000 (9)** |  |
| Pharmacotherapy | 4 | 0.098 | 0.176 | 0.555 | .579 |  |  |
| Technology | 1 | -0.146 | 0.433 | -0.337 | .736 |  |  |
| Psychotherapy | 4 | 0.178 | 0.169 | 1.054 | .292 |  |  |
| **Psychoeducation_TBI** | 3 | -0.266 | 0.232 | -1.146 | .252 | **0.000 (3)** |  |
| Pharmacotherapy | 1 | -0.159 | 0.555 | -0.286 | .775 |  |  |
| Technology | 0 | - | - | - | - |  |  |
| Psychotherapy | 2 | -0.289 | 0.256 | -1.130 | .259 |  |  |
| **Social cognition_TBI** |  |  |  |  |  |  |  |
| Psychotherapy | **2** | **0.090** | **0.258** | **0.350** | **.726** | **0.000 (2)** |  |
| *Two therapies* |  |  |  |  |  |  |  |
| **CBT_TBI +  Vocational therapy_TBI** |  |  |  |  |  |  |  |
| Psychotherapy | 1 | -0.321 | 0.412 | -0.780 | .436 | 0.000 (1) |  |
| **CBT_TBI + Cognitive training_TBI** |  |  |  |  |  |  |  |
| Pharmacotherapy | 2 | -0.127 | 0.247 | -0.514 | .607 | 0.000 (2) |  |
| **Cognitive training_TBI + Psychoeducation_TBI** |  |  |  |  |  |  |  |
| Psychotherapy | 1 | 0.086 | 0.223 | 0.385 | .700 | 0.000 (1) |  |
| **Cognitive training_TBI +  Social cognition_TBI** | **5** | **-0.241** | **0.286** | **-0.843** | **.399** | **0.331 (5)** |  |
| Pharmacotherapy | 1 | 0.241 | 0.628 | 0.383 | .702 |  |  |
| Psychotherapy | 4 | -0.380 | 0.335 | -1.133 | .257 |  |  |
| *Three therapies* |  |  |  |  |  |  |  |
| **Cognitive training_TBI + CBT_TBI + Vocational therapy_TBI**  Psychotherapy | 1 | -0.090 | 0.418 | -0.214 | .830 | 0.000 (1) |  |

*Notes*. k= number of effect sizes within each category; *g* = overall effect size; SE= standard error;= between-studies variance. In this column, the number of studies analyzed is indicated in brackets;= between-outcomes variance. In this column, the number of effect sizes analyzed is indicated in brackets.

| **eTable 4. Negative symptomatology results. Meta-analytic estimates for each therapy (or combination of therapies) and for each type of control group.** | | | | | | | |
| --- | --- | --- | --- | --- | --- | --- | --- |
| **NEGATIVE SYMPTOMATOLOGY** | | | | | | | |
| **Types of intervention** | *k* | *g* | SE | Z | *p*-value |  |  |
| **Total_TBI** | 23 | 0.081 | 0.078 | 1.031 | .302 | 0.019 (18) | 0.000 (23) |
|  |  |  |  |  |  |  |  |
| *One therapy* |  |  |  |  |  |  |  |
| **CBT_TBI** |  |  |  |  |  |  |  |
| Psychotherapy | 1 | 0.906 | 0.447 | 2.027 | .043 | 0.000 (1) |  |
| **Cognitive** **training_TBI** | **8** | **0.091** | **0.130** | **0.701** | **.483** | **0.004 (8)** |  |
| Pharmacotherapy | 2 | 0.028 | 0.305 | 0.093 | .926 |  |  |
| Technology | 1 | -0.151 | 0.475 | -0.319 | .750 |  |  |
| Psychotherapy | 5 | 0.175 | 0.179 | 0.976 | .329 |  |  |
| **Psychoeducation_TBI** | 3 | -0.516 | 0.227 | -2.269 | .023 | 0.000 (3) |  |
| Pharmacotherapy | 1 | -0.304 | 0.527 | -0.575 | .565 |  |  |
| Technology | 0 | - | - | - | - |  |  |
| Psychotherapy | 2 | -0.564 | 0.252 | -2.240 | .025 |  |  |
| **Social cognition_TBI** |  |  |  |  |  |  |  |
| Psychotherapy | 3 | 0.305 | 0.221 | 1.381 | .167 | 0.000 (3) |  |
| *Two therapies* |  |  |  |  |  |  |  |
| **CBT_TBI +  Vocational therapy_TBI** |  |  |  |  |  |  |  |
| Psychotherapy | 1 | -0.551 | 0.422 | -1.304 | .192 | 0.000 (1) |  |
| **Cognitive training_TBI + Psychoeducation_TBI** |  |  |  |  |  |  |  |
| Psychotherapy | 1 | 0.131 | 0.222 | 0.588 | .556 | 0.000 (1) |  |
| **Cognitive training_TBI +  Social cognition_TBI** | 5 | 0.127 | 0.121 | 1.050 | .294 | 0.000 (5) |  |
| Pharmacotherapy | 1 | 0.161 | 0.205 | 0.786 | .432 |  |  |
| Psychotherapy | 4 | 0.108 | 0.157 | 0.687 | .492 |  |  |
| *Three therapies* |  |  |  |  |  |  |  |
| **Cognitive training_TBI + CBT_TBI + Vocational therapy_TBI**  Psychotherapy | 4 | 0.108 | 0.157 | 0.687 | .492 | 0.000 (1) |  |

*Notes*. k= number of effect sizes within each category; *g* = overall effect size; SE= standard error;= between-studies variance. In this column, the number of studies analyzed is indicated in brackets;= between-outcomes variance. In this column, the number of effect sizes analyzed is indicated in brackets.

**eTable 5. Functioning results. Meta-analytic estimates for each therapy (or combination of therapies) and for each type of control group.**

| **FUNCTIONING** | | | | | | | |
| --- | --- | --- | --- | --- | --- | --- | --- |
| **Types of intervention** | k | *g* | SE | Z | *p*-value |  |  |
| **Total_TBI** | 98 | 0.245 | 0.090 | 2.724 | .006 | 0.057 (23) | 0.274 (98) |
|  |  |  |  |  |  |  |  |
| *One therapy* |  |  |  |  |  |  |  |
| **CBT_TBI** | 8 | 0.087 | 0.108 | 0.809 | .418 | 0.000 (2) |  |
| Pharmacotherapy | 1 | 0.548 | 0.402 | 1.363 | .173 |  |  |
| Psychotherapy | 11 | 0.052 | 0.112 | 0.462 | .644 |  |  |
| **Cognitive training_TBI** | 21 | 0.085 | 0.148 | 0.578 | .563 | 0.011 (11) | 0.291 (21) |
| Pharmacotherapy | 3 | -0.077 | 0.421 | -0.182 | .855 |  |  |
| Technology | 8 | -0.014 | 0.241 | -0.059 | .953 |  |  |
| Psychotherapy | 10 | 0.241 | 0.222 | 1.084 | .278 |  |  |
| **Psychoeducation_TBI**  Psychotherapy | 7 | 0.310 | 0.074 | 4.181 | <.0001 | 0.000 (2) |  |
| **Social cognition_TBI**  Psychotherapy | 8 | 0.554 | 0.119 | 4.665 | <.0001 | 0.004 (2) |  |
| *Two therapies* |  |  |  |  |  |  |  |
| **CBT_TBI +  Cognitive training_TBI** | 10 | 0.199 | 0.109 | 1.826 | .067 | 0.000 (2) |  |
| Pharmacotherapy | 3 | -0.090 | 0.205 | -0.441 | .659 |  |  |
| Psychotherapy | 7 | 0.318 | 0.133 | 2.384 | <.05 |  |  |
| **Cognitive training_TBI + Psychoeducation_TBI** |  |  |  |  |  |  |  |
| Psychotherapy | 2 | 0.374 | 0.255 | 1.465 | .143 | 0.078 (2) |  |
| **Cognitive training_TBI + Social cognition_TBI** | 36 | 0.549 | 0.265 | 2.073 | .038 | 0.097 (4) | 0.944 (36) |
| Pharmacotherapy | 2 | -0.023 | 0.754 | -0.030 | .976 |  |  |
| Psychotherapy | 34 | 0.654 | 0.258 | 2.530 | .011 |  |  |
| *Three therapies* |  |  |  |  |  |  |  |
| **Cognitive training_TBI + CBT_TBI +  Vocational therapy_TBI**  Psychotherapy | 2 | 0.147 | 0.209 | 0.702 | .483 | 0.000 (2) |  |

*Notes*. k= number of effect sizes within each category; *g* = overall effect size; SE= standard error;= between-studies variance. In this column, the number of studies analyzed is indicated in brackets;= between-outcomes variance. In this column, the number of effect sizes analyzed is indicated in brackets.

**eTable 6. Social cognition results. Meta-analytic estimates for each therapy (or combination of therapies) and for each type of control group.**

| **SOCIAL COGNITION** | | | | | | | |
| --- | --- | --- | --- | --- | --- | --- | --- |
| **Types of intervention** | *k* | *g* | SE | Z | *p*-value |  |  |
| **Total_TBI** | 44 | 0.317 | 0.129 | 2.459 | <.05 | 0.000 (17) | 0.596 (44) |
|  |  |  |  |  |  |  |  |
| *One therapy* |  |  |  |  |  |  |  |
| **CBT_TBI** |  |  |  |  |  |  |  |
| Psychotherapy | 1 | 0.055 | 0.339 | 0.162 | .871 |  |  |
| **Cognitive training_TBI** | **7** | **0.123** | **0.203** | **0.604** | **.546** | **0.143 (5)** |  |
| Pharmacotherapy | 2 | -0.395 | 0.248 | -1.588 | .112 |  |  |
| Technology | 3 | 0.598 | 0.203 | 2.948 | .003 |  |  |
| Psychotherapy | 2 | -0.164 | 0.323 | -0.508 | .611 |  |  |
| **Social cognition_TBI** |  |  |  |  |  |  |  |
| Psychotherapy | 4 | 0.277 | 0.226 | 1.224 | .221 | 0.075 (4) |  |
| *Two therapies* |  |  |  |  |  |  |  |
| **CBT_TBI + Cognitive training_TBI** |  |  |  |  |  |  |  |
| Psychotherapy | 2 | 0.254 | 0.250 | 1.015 | .310 | 0.000 (2) |  |
| **Cognitive training_TBI + Psychoeducation_TBI** |  |  |  |  |  |  |  |
| Psychotherapy | 1 | 0.267 | 0.221 | 1.208 | .227 |  |  |
| **Cognitive training_TBI + Social cognition_TBI** | **27** | **0.409** | **0.223** | **1.837** | **.066** | **0.019 (5)** | **1.026 (27)** |
| Pharmacotherapy | 1 | 0.349 | 1.010 | 0.317 | .751 |  |  |
| Technology | 1 | 0.374 | 1.204 | 0.310 | .756 |  |  |
| Psychotherapy | 25 | 0.441 | 0.270 | 1.630 | .103 |  |  |
| *Three therapies* |  |  |  |  |  |  |  |
| **Cognitive training_TBI + CBT_TBI + Vocational therapy_TBI**  Pharmacotherapy | 2 | 0.316 | 0.219 | 1.443 | .149 | 0.000 (2) |  |

*Notes*. k= number of effect sizes within each category; *g* = overall effect size; SE= standard error;= between-studies variance. In this column, the number of studies analyzed is indicated in brackets;= between-outcomes variance. In this column, the number of effect sizes analyzed is indicated in brackets.

**eTable 7. Quality of life results. Meta-analytic estimates for each therapy (or combination of therapies) and for each type of control group.**

| **QUALITY OF LIFE** | | | | | | | |
| --- | --- | --- | --- | --- | --- | --- | --- |
| **Types of intervention** | *k* | *g* | SE | Z | *p*-value |  |  |
| **Total_TBI** | 38 | 0.142 | 0.080 | 1.778 | .076 | 0.046 (16) | 0.000 (38) |
|  |  |  |  |  |  |  |  |
| *One therapy* |  |  |  |  |  |  |  |
| **Cognitive training_TBI** | **14** | **0.172** | **0.159** | **1.084** | **.278** | **0.108 (7)** | **0.000 (14)** |
| Pharmacotherapy | 2 | 0.035 | 0.317 | 0.111 | .911 |  |  |
| Technology | 9 | 0.300 | 0.215 | 1.393 | .164 |  |  |
| Psychotherapy | 2 | -0.267 | 0.544 | -0.492 | .633 |  |  |
| **Psychoeducation_TBI** |  |  |  |  |  |  |  |
| Psychotherapy | 9 | 0.024 | 0.084 | 0.288 | .773 | 0.000 (2) |  |
| **Social cognition_TBI** |  |  |  |  |  |  |  |
| Psychotherapy | 4 | 0.348 | 0.201 | 1.732 | .083 | 1 |  |
| **CBT _TBI** |  |  |  |  |  |  |  |
| Psychotherapy | 1 | 1.235 | 0.476 | 2.594 | <.01 | 1 |  |
| *Two therapies* |  |  |  |  |  |  |  |
| **CBT_TBI +  Cognitive training_TBI** | **6** | **0.016** | **0.168** | **0.097** | **.923** | **0.045 (2)** |  |
| Pharmacotherapy | 2 | -0.292 | 0.268 | -1.091 | .275 |  |  |
| Psychotherapy | 4 | 0.170 | 0.189 | 0.897 | .379 |  |  |
|  |  |  |  |  |  |  |  |
| **Cognitive training_TBI + Social cognition_TBI** | **4** | **0.044** | **0.150** | **0.292** | **.770** | **0.000 (4)** |  |
| Technology | 1 | -0.277 | 0.530 | -0.529 | .601 |  |  |
| Psychotherapy | 3 | 0.072 | 0.156 | 0.459 | .646 |  |  |

*Notes*. k= number of effect sizes within each category; *g* = overall effect size; SE= standard error;= between-studies variance. In this column, the number of studies analyzed is indicated in brackets;= between-outcomes variance. In this column, the number of effect sizes analyzed is indicated in brackets.

**eAppendix 5. Results from the network meta-analysis and list of interventions that work best for each outcome. At a general level, according to age and according to diagnosis**

For the network meta-analyses, we carried out the following analyses for each domain. First, all studies were analyzed together. Second, studies were divided based on the mean age of the participants: mean age above 25 and below 25. This cutoff is based on previous studies with a similar sample^2-4^. Two separate network meta-analyses were applied on each subset of studies. There were only a few studies where the mean age of the participants was below 25, so only the subset of studies with a mean age over 25 was analyzed. Third, studies were divided based on the diagnosis of the participants: First episode psychosis (FEP) (i.e., studies that labeled their patients as first episode psychosis, psychosis / high risk of psychosis*, psychotic disorder and one that spoke of "early psychosis.") and Schizophrenia (i.e., made up of those studies that labeled their patients as schizophrenia, schizophrenia / another psychotic disorder, schizophrenia / psychosis, schizophrenia / schizoaffective, schizophrenia / schizoaffective / psychosis, schizophrenia / schizophreniform / schizoaffective and non-affective psychosis).

* All the studies that have been included had at least 60% of the sample met criteria for psychosis.

**Neurocognition**

Results from the network meta-analysis (including all studies)

In total, there are 351 comparisons within this outcome. The mean age of this subsample is 37.75, with a standard deviation of 9.42. Thirty-four comparisons come from a sample of participants that have had a first episode of psychosis, whereas 317 comparisons come from samples of participants with schizophrenia. For the results of the P-Scores below, the reference category is the control group Technology only. Interventions are ordered by the P-score ranks.

**eTable 8. Results of the P-Scores for Neurocognition for all studies.**

| **Interventions** | **SMC and 95% CI** | **P-score** |
| --- | --- | --- |
| Cognitive training_TBI + Vocational therapy_TBI | 0.293 [-0.023, 0.609] | 0.9379 |
| Cognitive training_ TBI | 0.161 [0.093, 0.228] | 0.8744 |
| Cognitive training_TBI + Social cognition_TBI | 0.090 [-0.055, 0.234] | 0.6965 |
| Cognitive training_TBI + Psychoeducation_TBI | 0.058 [-0.179, 0.294] | 0.5856 |
| Pharmacotherapy | 0.051 [-0.073, 0.175] | 0.5759 |
| Cognitive training_TBI + CBT_TBI + Vocational therapy_TBI | 0.045 [-0.124, 0.215] | 0.5656 |
| Cognitive training_TBI + CBT_TBI | 0.042 [ -0.172, 0.257] | 0.5464 |
| Technology | - | 0.4151 |
| Social cognition_TBI | -0.034 [ -0.244, 0.176] | 0.3480 |
| Psychotherapy | -0.053 [ -0.174, 0.070] | 0.2500 |
| CBT_TBI + Vocational therapy_TBI | -0.098 [ -0.309, 0.113] | 0.2045 |
| CBT_TBI | -0.594 [ -0.874, -0.314] | 0.0001 |

*Notes*. SMC = standardized mean change; CI = confidence intervals.

The node-splitting method showed significant differences between the direct effect of cognitive training on control group – Psychotherapy (= 0.278) and the indirect effect obtained for the same pair of treatments (= 0.001, Z=2.248, *p* = .025).

**eFigure 2. Forest plot for relative treatment effectiveness for Neurocognition for all studies (351 comparisons).**

CG = control group.

Results from the network meta-analysis Including only studies where the mean age was over 25

There are 302 comparisons from samples whose mean age is over 25. Specifically, the mean age of this subsample is 39.78, with a standard deviation of 7.47. All of these comparisons come from samples of participants with schizophrenia. For the results of the P-Scores below, the reference category is the control group Technology only. Interventions are ordered by the P-score ranks.

**eTable 9. Results of the P-Scores for Neurocognition for studies where mean age of participants is over 25.**

| **Interventions** | **SMC and 95% CI** | **P-score** |
| --- | --- | --- |
| Cognitive training_TBI + Vocational therapy_TBI | 0.295 [-0.039, 0.629] | 0.9327 |
| Cognitive training_TBI | 0.174 [0.096, 0.251] | 0.8834 |
| Cognitive training_TBI + Social cognition_TBI | 0.094 [-0.067, 0.256] | 0.6994 |
| Pharmacotherapy | 0.062 [-0.070, 0.195] | 0.6016 |
| Cognitive training_TBI + CBT_TBI + Vocational therapy_TBI | 0.049 [-0.141, 0.238] | 0.5698 |
| Cognitive training_TBI + CBT_TBI | 0.045 [-0.184, 0.275] | 0.5509 |
| Technology | . | 0.4144 |
| Social cognition_TBI | -0.028 [-0.251, 0.195] | 0.3651 |
| Psychotherapy | -0.059 [-0.196, 0.096] | 0.2655 |
| CBT_TBI + Vocational therapy_TBI | -0.096 [-0.325, 0.134] | 0.2171 |
| CBT_TBI | -0.591 [-0.887,-0.295] | 0.0002 |

*Notes*. SMC = standardized mean change; CI = confidence intervals.

Although this subsample of studies is more homogenous in terms of the mean age of the participants and their diagnosis, the node-splitting method showed again significant differences between the direct effect of cognitive training on control group – Psychotherapy (= 0.332) and the indirect effect obtained for the same pair of treatments (= 0.004, Z=2.422, *p* = .015).

**eFigure 3. Forest plot of the relative treatment effectiveness for Neurocognition for studies where mean age of participants is over 25 (*k=*302).**

CG = control group.

**eFigure 4. Network plot for the interventions for Neurocognition (studies where mean age of participants is over 25, *k=*302).**

The thickness of the lines is proportional to the number of studies that reported the comparison between those treatments. CG = control group.

Results from the network meta-analysis including only studies where participants have a diagnosis of First Episode Psychosis

There are 34 comparisons from samples of participants that had a first episode of psychosis. The mean age of this subsample is 19.18, with a standard deviation of 4.65. For the results of the P-Scores below, the reference category is the control group Technology only. Interventions are ordered by the P-score ranks.

**eTable 10. Results of the P-Scores for Neurocognition for studies where participants have a diagnosis of First Episode Psychosis.**

| **Interventions** | **SMC and 95% CI** | **P-score** |
| --- | --- | --- |
| Technology | . | 0.7087 |
| Cognitive training_TBI | -0.011 [-0.187, 0.166] | 0.6894 |
| Cognitive training_TBI + Psychoeducation_TBI | -0.079 [-0.411, 0.253] | 0.5171 |
| Psychotherapy | -0.189 [-0.480, 0.102] | 0.0848 |

*Notes*. SMD = standardized mean change; CI = confidence intervals.

No inconsistent effects were detected

**eFigure 5. Forest plot of the relative treatment effectiveness for Neurocognition for studies where participants have a diagnosis of First Episode Psychosis (*k*=34).**

CG = control group.

**eFigure 6. Network plot for the interventions for Neurocognition (studies where participants have a diagnosis of First Episode Psychosis (*k*=34).**

The thickness of the lines is proportional to the number of studies that reported the comparison between those treatments. CG = control group.

Results from the network meta-analysis Including only studies where participants have a diagnosis of schizophrenia

There are 317 comparisons from samples of participants that have schizophrenia. The mean age of this subsample is 39.30, with a standard deviation of 7.94. For the results of the P-Scores below, the reference category is the control group Technology only. Interventions are ordered by the P-score ranks.

**eTable 11. Results of the P-Scores for Neurocognition for studies where participants have a diagnosis of schizophrenia.**

| **Interventions** | **SMC and 95% CI** | **P-score** |
| --- | --- | --- |
| Cognitive training_TBI + Vocational therapy_TBI | 0.310 [-0.017, 0.636] | 0.9374 |
| Cognitive training_TBI | 0.182 [0.109, 0.254] | 0.8811 |
| Cognitive training_TBI + Social cognition_TBI | 0.107 [-0.046, 0.261] | 0.7055 |
| Pharmacotherapy | 0.072 [-0.057, 0.200] | 0.5985 |
| Cognitive training_TBI + CBT_TBI + Vocational therapy_TBI | 0.063 [-0.117, 0.242] | 0.5795 |
| Cognitive training_TBI + CBT_TBI | 0.059 [-0.164, 0.282] | 0.5574 |
| Technology | - | 0.3826 |
| Social cognition_TBI | -0.016 [-0.233, 0.201] | 0.3668 |
| Psychotherapy | -0.036 [-0.170, 0.098] | 0.2712 |
| CBT_TBI + Vocational therapy_TBI | -0.081 [-0.302, 0.140] | 0.2199 |
| CBT_TBI | -0.577 [-0.866, -0.288] | 0.0001 |

*Notes*. SMD = standardized mean change; CI = confidence intervals.

Although this subsample of studies is more homogenous in terms of the participants diagnosis, the node-splitting method showed again significant differences between the direct effect of cognitive training on control group – Psychotherapy (= 0.298) and the indirect effect obtained for the same pair of treatments (= 0.004, Z=2.95, *p* = .023).

**eFigure 7. Forest plot of the relative treatment effectiveness for Neurocognition for studies where participants have a diagnosis of schizophrenia (*k*=317).**

CG = control group.

**eFigure 8. Network plot for the interventions for Neurocognition (studies where participants have a diagnosis of schizophrenia, *k* = 317).**

The thickness of the lines is proportional to the number of studies that reported the comparison between those treatments. CG = control group.

**Symptomatology**

Results from the network meta-analysis (including all studies)

In total, there are 130 comparisons within this outcome. The mean age of this subsample is 35.91, with a standard deviation of 9.46. Nineteen comparisons come from a sample of participants that have had a first episode of psychosis, whereas 98 comparisons come from samples of participants with schizophrenia. For the results of the P-Scores below, the reference category is the control group Technology only. Interventions are ordered by the P-score ranks.

**eTable 12. Results of the P-Scores for Symptomatology for all studies.**

| **Intervention** | **SMC and 95% CI** | **P-score** |
| --- | --- | --- |
| CBT_TBI | 0.105 [-0.353, 0.563] | 0.8419 |
| Cognitive training_TBI + Social cognition_TBI | 0.038 [-0.408, 0.484] | 0.7598 |
| Technology | - | 0.6946 |
| Cognitive training_TBI | -0.038 [-0.382, 0.306] | 0.6518 |
| Pharmacotherapy | -0.063 [-0.488, 0.361] | 0.6071 |
| Social cognition_TBI | -0.089 [-0.563, 0.386] | 0.5750 |
| Cognitive training_TBI + Psychoeducation_TBI | -0.166 [-0.887, 0.556] | 0.4849 |
| Cognitive training_TBI + CBT_TBI + Vocational therapy_TBI | -0.186 [-0.809, 0.437] | 0.4629 |
| Cognitive training_TBI + CBT_TBI | -0.251 [-0.903, 0.402] | 0.3859 |
| Psychotherapy | -0.301 [ -0.683, 0.081] | 0.2675 |
| Psychoeducation_TBI | -0.444 [-0.849, -0.038] | 0.1431 |
| CBT_TBI + Vocational therapy_TBI | -0.540 [-1.200, 0.120] | 0.1254 |

*Notes*. SMD = standardized mean change; CI = confidence intervals.

The node-splitting method showed significant differences between the direct effect of CBT on control group – Pharmacotherapy (= -0.10) and the indirect effect obtained for the same pair of treatments (= 0.64, Z= -2.34, *p* = .019). Also, significant differences were found between the overall direct and indirect effect of CB treatment on control group – Psychotherapy (= 0.66;= -0.08, Z= 2.34, *p* = .019). Lastly, inconsistency was also found between Psychoeducation and CG-Psychotherapy (= -0.40;= 0.29, Z= -2.18, *p* = .030).

**eFigure 9. Forest plot of the relative treatment effectiveness for Symptomatology with all studies included *(k*=130).**

CG = control group.

Results from the network meta-analysis Including only studies where the mean age was over 25

There are 101 comparisons from samples whose mean age is over 25. Specifically, the mean age of this subsample is 39.04, with a standard deviation of 6.51. All these comparisons came from samples diagnosed with schizophrenia. For the results of the P-Scores below, the reference category is the control group Technology only. Interventions are ordered by the P-score ranks.

**eTable 13. Results of the P-Scores for Symptomatology for studies where the mean age was over 25.**

| **Interventions** | **SMC and 95% CI** | **P-score** |
| --- | --- | --- |
| Cognitive training_TBI + Social cognition_TBI | 0.286 [-0.321, 0.893] | 0.8711 |
| Social cognition_TBI | 0.163 [-0.473, 0.799] | 0.7164 |
| Cognitive training_TBI | 0.138 [0.415, 0.691] | 0.7044 |
| Cognitive training_TBI + CBT_TBI + Vocational therapy_TBI | 0.102 [-0.662, 0.866] | 0.6247 |
| Technology | - | 0.5055 |
| Psychotherapy | -0.013 [-0.574, 0.548] | 0.4647 |
| CBT_TBI | -0.048 [-0.789, 0.692] | 0.4474 |
| Pharmacotherapy | -0.041 [-0.638, 0.556] | 0.4392 |
| Cognitive training_TBI + CBT_TBI | -0.153 [-0.948, 0.641] | 0.3401 |
| CBT_TBI + Vocational therapy_TBI | -0.252 [-1.049, 0.545] | 0.2378 |
| Psychoeducation_TBI | -0.286 [-0.810, 0.238] | 0.1489 |
| *Notes*. SMD = standardized mean change; CI = confidence intervals. | |  |

There are no inconsistent effects.

**eFigure 10. Forest plot of the relative treatment effectiveness for Symptomatology for the subset of studies where the man age was over 25 (*k*=101).**

CG = control group.

**eFigure 11. Network plot for the interventions for Symptomatology for the subset of studies where the man age was over 25 (*k*=101).**

The thickness of the lines is proportional to the number of studies that reported the comparison between those treatments. CG = control group.

Results from the network meta-analysis including only studies where participants have a diagnosis of First Episode Psychosis

There are 19 comparisons within this subgroup. The mean age is 19.19 and the standard deviation is 3.12. For the results of the P-Scores below, the reference category is the control group Technology only. Interventions are ordered by the P-score ranks.

**eTable 14. Results of the P-Scores for Symptomatology for studies where participants were diagnosed with First Episode Psychosis.**

| **Interventions** | **SMC and 95% CI** | **P-score** |
| --- | --- | --- |
| CBT_TBI | 0.756 [0.094, 1.418] | 0.9965 |
| Cognitive training_TBI + Psychoeducation_TBI | 0.265 [-0.413, 0.943] | 0.6483 |
| Psychotherapy | 0.129 [-0.508, 0.766] | 0.4182 |
| Technology | - | 0.3869 |
| Cognitive training_TBI | -0.238 [0.491, 0.014] | 0.0501 |

*Notes*. SMD = standardized mean change; CI = confidence intervals.

Consistency could not be assessed because for each pair of treatments, only direct or indirect evidence was available.

**eFigure 12. Forest plot of the relative treatment effectiveness for Symptomatology for the subset of studies where participants were diagnosed with First Episode Psychosis (*k*=19).**

CG = control group.

**eFigure 13. Network plot for the interventions for Symptomatology for the subset of studies where participants were diagnosed with First Episode Psychosis (*k*=19).**

The thickness of the lines is proportional to the number of studies that reported the comparison between those treatments. CG = control group.

Results from the network meta-analysis including only studies where participants have a diagnosis schizophrenia

There are 111 comparisons for this subgroup, whose mean age is 38.86 and the standard deviation is 6.71. For the results of the P-Scores below, the reference category is the control group Technology only. Interventions are ordered by the P-score ranks.

**eTable 15. Results of the P-Scores for Symptomatology for studies where participants were diagnosed with schizophrenia.**

| **Intervention** | **SMC and 95% CI** | **P-score** |
| --- | --- | --- |
| Cognitive training_TBI + Social cognition_TBI | 0.224 [-0.330, 0.778] | 0.8508 |
| Cognitive training_TBI | 0.161 [-0.325, 0.648] | 0.7906 |
| Social cognition_TBI | 0.123 [-0.461, 0.708] | 0.7161 |
| Cognitive training_TBI + CBT_TBI + Vocational therapy_TBI | 0.032 [-0.685, 0.748] | 0.5913 |
| Technology | - | 0.5517 |
| Pharmacotherapy | -0.055 [-0.603, 0.494] | 0.4814 |
| Psychotherapy | -0.084 [-0.586, 0.419] | 0.4200 |
| Cognitive training_TBI + CBT_TBI | -0.149, [-0.898, 0.601] | 0.3846 |
| CBT_TBI | -0.157 [-0.828, 0.513] | 0.3579 |
| CBT_TBI + Vocational therapy_TBI | -0.323 [-1.074, 0.429] | 0.2090 |
| Psychoeducation_TBI | -0.326 [-0.811, 0.159] | 0.1467 |

*Notes*. SMD = standardized mean change; CI = confidence intervals.

There were no inconsistent effects.

**eFigure 14. Forest plot of the relative treatment effectiveness for Symptomatology for the subset of studies where participants were diagnosed with schizophrenia (*k*=111).**

CG = control group.

**eFigure 15. Network plot for the interventions for Symptomatology for the subset of studies where participants were diagnosed with schizophrenia (*k*=111).**

The thickness of the lines is proportional to the number of studies that reported the comparison between those treatments. CG = control group.

**Functioning**

Results from the network meta-analysis (including all studies)

In total, there are 108 comparisons within this outcome. The mean age of this subsample is 36.58, with a standard deviation of 10.66. Nine comparisons come from a sample of participants that have had a first episode of psychosis, whereas 97 comparisons come from samples of participants with schizophrenia. For the results of the P-Scores below, the reference category is the control group Technology only. Interventions are ordered by the P-score ranks.

**eTable 16. Results of the P-Scores for Functioning for all studies.**

| **Intervention** | **SMC and 95% CI** | **P-score** |
| --- | --- | --- |
| Cognitive training_TBI + Social cognition_TBI | 0.390 [-0.002, 0.782] | 0.9470 |
| Social cognition_TBI | 0.294 [-0.158, 0.747] | 0.8513 |
| Cognitive training_TBI + Psychoeducation_TBI | 0.106 [-0.553, 0.765] | 0.5964 |
| Pharmacotherapy | 0.046 [-0.390, 0.481] | 0.5533 |
| Technology | - | 0.4754 |
| Psychoeducation_TBI | -0.007 [-0.477, 0.463] | 0.4663 |
| Cognitive training_TBI | -0.013 [-0.297, 0.272] | 0.4572 |
| CBT_TBI | -0.032 [-0.459, 0.397] | 0.4308 |
| Cognitive training_TBI + CBT_TBI + Vocational therapy_TBI | -0.112 [-0.778, 0.554] | 0.3451 |
| Cognitive training_TBI + CBT_TBI | -0.100 [-0.526, 0.327] | 0.3113 |
| Psychotherapy | -0.268 [-0.636, 0.101] | 0.0657 |

*Notes*. SMD = standardized mean change; CI = confidence intervals.

The node-splitting method showed significant differences between the direct effect of CBT on control group – Pharmacotherapy (= -0.27) and the indirect effect obtained for the same pair of treatments (= 0.55, Z= 2.13, *p* = .033).

**eFigure 16. Forest plot of the relative treatment effectiveness for Functioning with all studies included (k=108).**

CG = control group.

Results from the network meta-analysis Including only studies where the mean age was over 25

There are 91 comparisons from samples whose mean age is over 25. Specifically, the mean age of this subsample is 38.57, with a standard deviation of 9.46. Only one from these comparisons came from a sample of participant a first episode of psychosis, while the remaining 90 comparisons came from samples diagnosed with schizophrenia. For the results of the P-Scores below, the reference category is the control group Technology only. Interventions are ordered by the P-score ranks.

**eTable 17. Results of the P-Scores for Functioning for studies where the mean age was over 25.**

| **Intervention** | **SMC and 95% CI** | **P-score** |
| --- | --- | --- |
| Cognitive training_TBI + Social cognition_TBI | 0.329 [-0.370, 1.027] | 0.9243 |
| Social cognition_TBI | 0.208 [-0.552, 0.967] | 0.7983 |
| Pharmacotherapy | -0.011 [-0.753, 0.731] | 0.5756 |
| Technology | - | 0.5479 |
| CBT_TBI | -0.035 [-0.793, 0.723] | 0.3282 |
| Cognitive training_TBI | -0.078 [-0.684, 0.528] | 0.4898 |
| Cognitive training_TBI + CBT_TBI | -0.150 [-0.886, 0.586] | 0.3978 |
| Cognitive training_TBI + CBT_TBI + Vocational therapy_TBI | -0.201 [-1.198, 0.796] | 0.3928 |
| Psychotherapy | -0.357 [-1.034, 0.321] | 0.1628 |
| Psychoeducation_TBI | -0.685 [-1.920, 0.550] | 0.1387 |

*Notes*. SMD = standardized mean change; CI = confidence intervals.

There were no inconsistent effects

**eFigure 17. Forest plot of the relative treatment effectiveness for Functioning for the subset of studies where the mean age was over 25 (*k*=91).**

CG = control group.

**eFigure 18. Network plot for the interventions for Functioning for the subset of studies where the mean age was over 25 (*k*=91).**

The thickness of the lines is proportional to the number of studies that reported the comparison between those treatments. CG = control group.

Results from the network meta-analysis including only studies where participants have a diagnosis of First Episode Psychosis

There are 9 comparisons within this subgroup. The mean age is 22.37 and the standard deviation is 8.51. For the results of the P-Scores below, the reference category is the control group Technology only. Interventions are ordered by the P-score ranks.

**eTable 18. Results of the P-Scores for Functioning for studies where participants were diagnosed with First Episode Psychosis.**

| **Intervention** | **SMC and 95% CI** | **P-score** |
| --- | --- | --- |
| Cognitive training_TBI + Psychoeducation_TBI | 0.174 [-0.953, 1.301] | 0.7192 |
| Cognitive training_TBI | 0.176 [-0.401, 0.753] | 0.6992 |
| Technology | - | 0.4784 |
| CBT_TBI | -0.149 [-1.232, 0.935] | 0.3499 |
| Psychotherapy | -0.199 [1.205, 0.806] | 0.2533 |

*Notes*. SMD = standardized mean change; CI = confidence intervals.

Consistency could not be assessed because for each pair of treatments, only direct or indirect evidence was available.

eFigure 19. Forest plot of the relative treatment effectiveness for Functioning for the subset of studies where participants were diagnosed with First Episode Psychosis (*k*=9).

CG = control group.

**eFigure 20. Network plot for the interventions for Functioning for the subset of studies where participants were diagnosed with First Episode Psychosis (*k*=9).**

The thickness of the lines is proportional to the number of studies that reported the comparison between those treatments. CG = control group.

Results from the network meta-analysis including only studies where participants have a diagnosis schizophrenia

There are 90 comparisons within this subgroup. The mean age is 37.96 and the standard deviation is 9.83. For the results of the P-Scores below, the reference category is the control group Technology only. Interventions are ordered by the P-score ranks.

**eTable 19. Results of the P-Scores for Functioning for studies where participants were diagnosed with schizophrenia.**

| **Intervention** | **SMC and 95% CI** | **P-score** |
| --- | --- | --- |
| Cognitive training_TBI + Social cognition_TBI | 0.342 [-0.085, 0.768] | 0.9532 |
| Social cognition_TBI | 0.244 [-0.238, 0.727] | 0.8454 |
| Pharmacotherapy | 0.017 [-0.450, 0.483] | 0.5602 |
| CBT_TBI | -0.001 [-0.483, 0.481] | 0.5331 |
| Technology | - | 0.5266 |
| Psychoeducation_TBI | -0.057 [-0.557, 0.443] | 0.4413 |
| Cognitive training_TBI | -0.065 [-0.388, 0.258] | 0.4198 |
| Cognitive training_TBI + CBT_TBI | -0.117 [-0.578, 0.344] | 0.3340 |
| Cognitive training_TBI + CBT_TBI + Vocational therapy_TBI | -0.162 [-0.852, 0.527] | 0.3292 |
| Psychotherapy | -0.318 [-0.723, 0.088] | 0.0571 |

*Notes*. SMD = standardized mean change; CI = confidence intervals.

The node-splitting method showed significant differences between the direct effect of CBT on control group – Pharmacotherapy (= 0.55) and the indirect effect obtained for the same pair of treatments (= -0.44, Z= 2.09, *p* = .034).

**eFigure 21. Forest plot of the relative treatment effectiveness for Functioning for the subset of studies where participants were diagnosed with schizophrenia (*k*=90).**

CG = control group.

**eFigure 22. Network plot for the interventions for Functioning for the subset of studies where participants were diagnosed with schizophrenia (*k*=90).**

The thickness of the lines is proportional to the number of studies that reported the comparison between those treatments. CG = control group.

**Social cognition**

Results from the network meta-analysis (including all studies)

In total, there are 46 comparisons within this outcome. The mean age of this subsample is 36.84, with a standard deviation of 8.69. Only one comparison come from a sample of participants that have had a first episode of psychosis, whereas 45 comparisons come from samples of participants with schizophrenia. For the results of the P-Scores below, the reference category is the control group Technology only. Interventions are ordered by the P-score ranks.

**eTable 20. Results of the P-Scores for Social cognition for all studies.**

| **Intervention** | **SMC and 95% CI** | **P-score** |
| --- | --- | --- |
| Cognitive training_TBI + Social cognition_TBI | 0.800 [-0.150, 1.749] | 0.7035 |
| Pharmacotherapy | 0.724 [-0.380, 1.827] | 0.6115 |
| Cognitive training_TBI + CBT_TBI + Vocational therapy_TBI | 0.721 [-0.667, 2.109] | 0.5959 |
| Social cognition_TBI | 0.690 [-0.430, 1.810] | 0.5937 |
| Cognitive training_TBI + CBT_TBI | 0.658 [-0.734, 2.050] | 0.5599 |
| Cognitive training_TBI + Psychoeducation_TBI | 0.671 [-1.038, 2.379] | 0.5539 |
| CBT_TBI | 0.459 [-1.185, 2.104] | 0.4434 |
| Cognitive training_TBI | 0.468 [-0.282, 1.218] | 0.4341 |
| Psychotherapy | 0.404 [-0.584, 1.356] | 0.3463 |
| Technology | - | 0.1578 |

*Notes*. SMD = standardized mean change; CI = confidence intervals.

There were no inconsistent effects.

**eFigure 23. Forest plot of the relative treatment effectiveness for Social cognition (all studies included, *k*=46).**

CG = control group.

Results from the network meta-analysis Including only studies where the mean age was over 25

There are 45 comparisons from samples whose mean age is over 25. Specifically, the mean age of this subsample is 37.10, with a standard deviation of 8.60. All these comparisons come from samples of participants diagnosed with schizophrenia. For the results of the P-Scores below, the reference category is the control group Technology only. Interventions are ordered by the P-score ranks.

**eTable 21. Results of the P-Scores for Social cognition for studies where the mean age was over 25.**

| **Intervention** | **SMC and 95% CI** | **P-score** |
| --- | --- | --- |
| Cognitive training_TBI + Social Cognition_TBI | 0.800 [-0.150, 1.749] | 0.7203 |
| Pharmacotherapy | 0.724 [-0.380, 1.827] | 0.6224 |
| Cognitive training_TBI + CBT_TBI + Vocational therapy_TBI | 0.721 [-0.667, 2.109] | 0.6051 |
| Social cognition_TBI | 0.690 [-0.430, 1.810] | 0.6042 |
| Cognitive training_TBI + CBT_TBI | 0.658 [-0.734, 2.050] | 0.5681 |
| CBT_TBI | 0.459 [-1.185, 2.104] | 0.4469 |
| Cognitive training_TBI | 0.468 [-0.282, 1.218] | 0.4380 |
| Psychotherapy | 0.404 [-0.548, 1.356] | 0.3450 |
| Technology | - | 0.1499 |

*Notes*. SMD = standardized mean change; CI = confidence intervals.

There were no inconsistent effects

**eFigure 24. Forest plot of the relative treatment effectiveness for Social cognition for the subset of studies where the mean age was over 25 (*k*=45).**

CG = control group.

**eFigure 25. Network plot for the interventions for Social cognition for the subset of studies where the mean age of the participants was over (*k*=45).**

The thickness of the lines is proportional to the number of studies that reported the comparison between those treatments. CG = control group.

Results from the network meta-analysis including only studies where participants have a diagnosis of First Episode Psychosis

There was only one effect size for this subgroup, so we could not run the network meta-analysis.

Results from the network meta-analysis including only studies where participants have a diagnosis schizophrenia

There are 45 comparisons within this subgroup. The mean age of these participants was 37.10, and the standard deviation was 8.60. For the results of the P-Scores below, the reference category is the control group Technology only. Interventions are ordered by the P-score ranks.

**eTable 22. Results of the P-Scores for Social cognition for studies where participants were diagnosed with schizophrenia.**

| **Intervention** | **SMC and 95% CI** | **P-score** |
| --- | --- | --- |
| Cognitive training_TBI + Social cognition_TBI | 0.800 [-0.150, 1.749] | 0.7203 |
| Pharmacotherapy | 0.724 [-0.380, 1.827] | 0.6224 |
| Cognitive training_TBI + CBT_TBI + Vocational therapy_TBI | 0.721 [-0.667, 2.109] | 0.6051 |
| Social cognition_TBI | 0.690 [-0.430, 1.810] | 0.6042 |
| Cognitive training_TBI + CBT_TBI | 0.658 [-0.734, 2.050] | 0.5681 |
| CBT_TBI | 0.459 [-1.185, 2.104] | 0.4469 |
| Cognitive training_TBI | 0.468 [-0.282, 1.218] | 0.4380 |
| Psychotherapy | 0.404 [0.548, 1.356] | 0.3450 |
| Technology | - | 0.1499 |

*Notes*. SMD = standardized mean change; CI = confidence intervals.

There were no inconsistent effects

**eFigure 26. Forest plot of the relative treatment effectiveness for Social cognition for the subset of studies where participants were diagnosed with schizophrenia (*k*=45).**

CG = control group.

**eFigure 27. Network plot for the interventions for Social cognition for the subset of studies where participants were diagnosed with schizophrenia (*k*=45).**

The thickness of the lines is proportional to the number of studies that reported the comparison between those treatments. CG = control group.

**Quality of Life**

Results from the network meta-analysis (including all studies)

In total, there are 40 comparisons within this outcome. The mean age of this subsample is 37.85, with a standard deviation of 6.72. Only one comparison come from a sample of participants that have had a first episode of psychosis, whereas 39 comparisons come from samples of participants with schizophrenia. For the results of the P-Scores below, the reference category is the control group Technology only. Interventions are ordered by the P-score ranks.

**eTable 23. Results of the P-Scores for Quality of life for all studies.**

| **Intervention** | **SMC and 95% CI** | **P-score** |
| --- | --- | --- |
| CBT_TBI | 1.266 [0.457, 2.076] | 0.9952 |
| Social cognition_TBI | 0.383 [-0.121, 0.887] | 0.7369 |
| Pharmacotherapy | 0.316 [-0.064, 0.697] | 0.6636 |
| Cognitive training_TBI | 0.296 [0.102, 0.491] | 0.6575 |
| Cognitive training_TBI + CBT_TBI | 0.124 [-0.256, 0.504] | 0.3853 |
| Cognitive training_TBI + Social cognition_TBI | 0.088 [-0.362, 0.539] | 0.3351 |
| Psychoeducation_TBI | 0.086 [-0.348, 0.520] | 0.3306 |
| Psychotherapy | 0.031 [-0.359, 0.421] | 0.2057 |
| Technology | - | 0.1901 |

*Notes*. SMD = standardized mean change; CI = confidence intervals.

The node-splitting method showed significant differences between the direct effect of Cognitive Training on control group – Psychotherapy (= -0.27) and the indirect effect obtained for the same pair of treatments (= -0.57, Z= 2.162, *p* = .031).

**eFigure 28. Forest plot of the relative treatment effectiveness for Quality of life**

**for all studies (*k*=40).**

CG = control group.

Results from the network meta-analysis Including only studies where the mean age was over 25

There are 37 comparisons within this subgroup of studies. The mean age of this subsample is 38.35 and the standard deviation is 6.04. All these comparisons come from samples of participants with schizophrenia. For the results of the P-Scores below, the reference category is the control group Technology only. Interventions are ordered by the P-score ranks.

**eTable 24. Results of the P-Scores for Quality of life for studies where the man age was over 25.**

| **Intervention** | **SMC and 95% CI** | **P-score** |
| --- | --- | --- |
| Social cognition_TBI | 0.398 [-0.158, 0.954] | 0.8236 |
| Pharmacotherapy | 0.321 [-0.108, 0.750] | 0.7272 |
| Cognitive training_TBI | 0.298 [0.078, 0.517] | 0.7205 |
| Psychoeducation_TBI | 0.141 [-0.354, 0.636] | 0.4592 |
| Cognitive training_TBI + CBT_TBI | 0.134 [-0.289, 0.556] | 0.4313 |
| Cognitive training_TBI + Social Cognition_TBI | 0.096 [-0.400, 0.592] | 0.3786 |
| Psychotherapy | 0.046 [-0.383, 0.474] | 0.2482 |
| Technology only | - | 0.2115 |

*Notes*. SMD = standardized mean change; CI = confidence intervals.

There were no inconsistent effects.

**eFigure 29. Forest plot of the relative treatment effectiveness for Quality of life for the subset of studies where the man age was over 25 (*k*=37).**

CG = control group.

**eFigure 30. Network plot for the interventions for Quality of life for the subset of studies where the mean age was over 25 (*k*=37).**

The thickness of the lines is proportional to the number of studies that reported the comparison between those treatments. CG = control group.

Results from the network meta-analysis including only studies where participants have a diagnosis of First Episode Psychosis

There was only one effect size for this subgroup, so we could not run the network meta-analysis.

Results from the network meta-analysis including only studies where participants have a diagnosis schizophrenia

There are 39 comparisons within this subgroup. The mean age of these participants was 38.35, and the standard deviation was 6.04. For the results of the P-Scores below, the reference category is the control group Technology only. Interventions are ordered by the P-score ranks.

**eTable 25. Results of the P-Scores for Quality of life for studies where participants were diagnosed with schizophrenia.**

| **Intervention** | **SMC and 95% CI** | **P-score** |
| --- | --- | --- |
| Social cognition_TBI | 0.383 [-0.121, 0.887] | 0.8403 |
| Pharmacotherapy | 0.316 [-0.064, 0.697] | 0.7567 |
| Cognitive training_TBI | 0.296 [0.102, 0.491] | 0.7503 |
| Cognitive training_TBI + CBT_TBI | 0.124 [-0.255, 0.504] | 0.4401 |
| Cognitive training_TBI + Social cognition_TBI | 0.088 [-0.362, 0.539] | 0.3828 |
| Psychoeducation_TBI | 0.086 [-0.348, 0.520] | 0.3777 |
| Psychotherapy | 0.404 [0.548, 1.356] | 0.2350 |
| Technology | - | 0.2171 |

*Notes*. SMD = standardized mean change; CI = confidence intervals.

The node-splitting method showed significant differences between the direct effect of Cognitive Training on control group – Psychotherapy (= -0.27) and the indirect effect obtained for the same pair of treatments (= -0.57, Z= 2.162, *p* = .031).

**eFigure 26. Forest plot of the relative treatment effectiveness for Quality of Life for the subset of studies where participants were diagnosed with schizophrenia (*k*=39).**

CG = control group.

**eFigure 27. Network plot for the interventions for Quality of Life for the subset of studies where participants were diagnosed with schizophrenia (*k*=39).**

The thickness of the lines is proportional to the number of studies that reported the comparison between those treatments. CG = control group.

**eAppendix 6. Results of the moderator analyses**

These tables contain the results of the moderator analyses (for those subsets of data were more than 20 effect sizes were available). Sometimes the three-level model did not converge (due to a small number of effect sizes). In those cases, a standard random-effects model was fitted, and the cell in the column of the between-outcomes variance was left empty.

**eTable 26. Results of the moderator analyses for Neurocognition. Subset of studies where the TBI intervention was Cogntive training_TBI.**

| **NEUROCOGNITION** | | | | | | | | | |
| --- | --- | --- | --- | --- | --- | --- | --- | --- | --- |
| **Intervention: Cognitive training** | | | | | | | | | |
|  | *k* | | *g* | | SE | Z | *p*-value |  |  |
| **% men in control group** |  | |  | |  |  |  | 0.027 (17) | 0.043 (152) |
| Pharmacotherapy | 40 | | 0.087 | | 0.105 | 0.834 | .404 |  |  |
| Technology | 88 | | 0.166 | | 0.071 | 2.350 | .019 |  |  |
| Psychotherapy | 24 | | 0.351 | | 0.132 | 2.651 | .008 |  |  |
| % men_control_group | 152 | | 0.001 | | 0.005 | 0.180 | .857 |  |  |
|  |  | |  | |  |  |  |  |  |
| **% men in intervention** |  | |  | |  |  |  | 0.024 (17) | 0.044 (152) |
| Pharmacotherapy | 40 | | 0.081 | | 0.102 | 0.794 | .427 |  |  |
| Technology | 88 | | 0.144 | | 0.072 | 1.989 | .047 |  |  |
| Psychotherapy | 24 | | 0.420 | | 0.147 | 2.855 | .004 |  |  |
| % men_inter_group | 152 | | 0.005 | | 0.005 | 0.913 | .361 |  |  |
|  |  | |  | |  |  |  |  |  |
| **Mean age control group** |  | |  | |  |  |  | 0.028 (19) | 0.036 (169) |
| Pharmacotherapy | 40 | | 0.094 | | 0.103 | 0.911 | .362 |  |  |
| Technology | 105 | | 0.198 | | 0.063 | 3.147 | .002 |  |  |
| Psychotherapy | 24 | | 0.343 | | 0.123 | 2.790 | .005 |  |  |
| mean_age_control | 169 | | 0.004 | | 0.005 | 0.697 | .486 |  |  |
|  |  | |  | |  |  |  |  |  |
| **Mean age intervention group** |  | |  | |  |  |  | 0.027 (19) | 0.037 (169) |
| Pharmacotherapy | 40 | | 0.091 | | 0.102 | 0.889 | .374 |  |  |
| Technology | 105 | | 0.196 | | 0.062 | 3.154 | .002 |  |  |
| Psychotherapy | 24 | | 0.329 | | 0.124 | 2.658 | .008 |  |  |
| mean_age_intervent | 169 | | 0.004 | | 0.006 | 0.675 | .500 |  |  |
|  |  | |  | |  |  |  |  |  |
| **Education control group** |  | |  | |  |  |  | 0.019 (17) | 0.042 (161) |
| Pharmacotherapy | 37 | | 0.075 | | 0.104 | 0.722 | .471 |  |  |
| Technology | 105 | | 0.156 | | 0.060 | 2.583 | .010 |  |  |
| Psychotherapy | 19 | | 0.274 | | 0.142 | 1.923 | .055 |  |  |
| Education control | 161 | | 0.030 | | 0.017 | 1.788 | .074 |  |  |
|  |  | |  | |  |  |  |  |  |
| **Education intervention group** | | |  | |  |  |  | 0.017 (17) | 0.042 (161) |
| Pharmacotherapy | 37 | | 0.059 | | 0.102 | 0.586 | .558 |  |  |
| Technology | 105 | | 0.150 | | 0.059 | 2.559 | .011 |  |  |
| Psychotherapy | 19 | | 0.276 | | 0.137 | 2.015 | .044 |  |  |
| Education intervention | 161 | | 0.034 | | 0.016 | 2.135 | .033 |  |  |
| **Intervention: Cognitive training (cont.)** | | | | | | | | | |
|  | *k* | | *g* | | SE | Z | *p*-value |  |  |
| **Type of medication** |  | |  | |  |  |  | 0.015 (18) | 0.040 (159) |
| Pharmacotherapy | 40 | | -0.005 | | 0.125 | -0.043 | .966 |  |  |
| Technology | 88 | | 0.148 | | 0.122 | 1.213 | .225 |  |  |
| Psychotherapy | 31 | | 0.203 | | 0.144 | 1.408 | .159 |  |  |
| Atypical antipsychotic | 25 | | -0.010 | | 0.130 | 0.077 | .939 |  |  |
| Typical antipsychotic | 33 | | 0.243 | | 0.145 | 1.679 | .093 |  |  |
| *Reference category: Antipsychotics (K=101). When the reference category was shifted to “Typical Antipsychotic”, no differences were found neither.* | | | | | | | | | |
|  |  | |  | |  |  |  |  |  |
| **Amount of medication control** | | |  | |  |  |  | 0.000 (6) | 0.180 (48) |
| Pharmacotherapy | 19 | | 0.211 | | 0.127 | 1.663 | .096 |  |  |
| Technology | 19 | | 0.284 | | 0.131 | 2.167 | .030 |  |  |
| Psychotherapy | 10 | | 0.593 | | 0.171 | 3.462 | .001 |  |  |
| Amount medication control |  | | -0.001 | | 0.001 | -1.246 | .213 |  |  |
|  |  | |  | |  |  |  |  |  |
| **Amount of medication intervention** | | |  | |  |  |  | 0.000 (6) | 0.178 (48) |
| Pharmacotherapy | 19 | | 0.163 | | 0.125 | 1.304 | .192 |  |  |
| Technology | 19 | | 0.295 | | 0.126 | 2.336 | .020 |  |  |
| Psychotherapy | 10 | | 0.663 | | 0.185 | 3.586 | .0003 |  |  |
| Amount medication interve. |  | | -0.001 | | 0.001 | -1.379 | .168 |  |  |
|  |  | |  | |  |  |  |  |  |
| **Duration of intervention** |  | |  | |  |  |  | 0.023 (21) | 0.034 (184) |
| Pharmacotherapy | 40 | | 0.089 | | 0.099 | 0.899 | .369 |  |  |
| Technology | 105 | | 0.191 | | 0.060 | 3.183 | .002 |  |  |
| Psychotherapy | 39 | | 0.290 | | 0.097 | 2.999 | .003 |  |  |
| Duration intervention |  | | 0.0003 | | 0.005 | 0.056 | .955 |  |  |
|  |  | |  | |  |  |  |  |  |
| **Frequency of the intervention** | | |  | |  |  |  | 0.020 (21) | 0.035 (184) |
| Pharmacotherapy | 40 | | 0.104 | | 0.099 | 1.053 | .292 |  |  |
| Technology | 105 | | 0.180 | | 0.059 | 3.054 | .002 |  |  |
| Psychotherapy | 39 | | 0.285 | | 0.093 | 3.056 | .002 |  |  |
| Frequency | 184 | | 0.013 | | 0.018 | 0.740 | .459 |  |  |
|  |  | |  | |  |  |  |  |  |
| **Diagnosis** |  | |  | |  |  |  | 0.020 (21) | 0.034 (184) |
| Pharmacotherapy | 40 | | -0.011 | | 0.170 | -0.605 | .545 |  |  |
| Technology | 105 | | 0.180 | | 0.140 | 0.129 | .898 |  |  |
| Psychotherapy | 39 | | 0.127 | | 0.152 | 0.840 | .401 |  |  |
| Schizophrenia | 159 | | 0.195 | | 0.144 | 1.352 | .176 |  |  |
| *Reference category Psychosis (k=24)* | | | | | |  |  |  |  |
|  |  | |  | |  |  |  |  |  |
| **Contact control group** |  | |  | |  |  |  | 0.024 (21) | 0.033 (184) |
| Pharmacotherapy | 40 | | 0.080 | | 0.100 | 0.802 | .423 |  |  |
| Technology | 105 | | 0.159 | | 0.064 | 2.468 | .014 |  |  |
| Psychotherapy | 30 | | 0.206 | | 0.111 | 1.860 | .063 |  |  |
| Others | 21 | | 0.243 | | 0.143 | 1.704 | .088 |  |  |
| Therapists | 17 | | 0.060 | | 0.161 | 0.372 | .710 |  |  |
| *Reference category: No contact (k=146). When the reference category was shifted to “Others”, no differences were found neither.* | | | | | | | | | |
| **Intervention: Cognitive training (cont.)** | | | | | | | | | |
|  | *k* | | *g* | | SE | Z | *p*-value |  |  |
| **Contact intervention group** |  | |  | |  |  |  | 0.025 (21) | 0.033 (184) |
| Pharmacotherapy | 49 | | 0.051 | | 0.104 | 0.495 | .621 |  |  |
| Technology | 105 | | 0.164 | | 0.065 | 2.518 | .012 |  |  |
| Psychotherapy | 39 | | 0.197 | | 0.121 | 1.632 | .103 |  |  |
| Others | 33 | | 0.170 | | 0.125 | 1.360 | .174 |  |  |
| Therapists | 17 | | 0.060 | | 0.164 | 0.367 | .714 |  |  |
| *Reference category: No contact (k=134). When the reference category was shifted to “Others”, no differences were found neither.* | | | | | | | | | |
|  |  | |  | |  |  |  |  |  |
| **Setting** |  | |  | |  |  |  | 0.023 (21) | 0.034 (184) |
| Pharmacotherapy | 40 | | 0.082 | | 0.102 | 0.800 | .424 |  |  |
| Technology | 105 | | 0.187 | | 0.062 | 3.025 | .003 |  |  |
| Psychotherapy | 39 | | 0.189 | | 0.095 | 3.035 | .002 |  |  |
| Clinic | 173 | | 0.047 | | 0.167 | 0.279 | .780 |  |  |
| *Reference category: Home (k=11). There are not effects within the category Clinic/Home* | | | | | | | | | |
|  |  | |  | |  |  |  |  |  |
| **eTable 27. Results of the moderator analyses for Neurocognition. Subset of studies where the TBI intervention was Cognitive training_TBI + Social cognition_TBI.** | | | | | | | | | |
| **Intervention: Cognitive training + Social cognition** | | | | | | | | | |
|  | *k* | | *g* | | SE | Z | *p*-value |  |  |
| **% men in control group** |  | |  | |  |  |  | 0.000 (5) | 0.095 (46) |
| Pharmacotherapy | 11 | | 0.552 | | 0.235 | 2.344 | .019 |  |  |
| Technology | 1 | | -0.020 | | 0.627 | -0.031 | .975 |  |  |
| Psychotherapy | 34 | | -0.117 | | 0.134 | -0.874 | .382 |  |  |
| % men_control_group | 46 | | 0.038 | | 0.020 | 1.923 | .054 |  |  |
|  |  | |  | |  |  |  |  |  |
| **% men in intervention group** |  | |  | |  |  |  | 0.000 (5) | 0.102 (46) |
| Pharmacotherapy | 11 | | -0.113 | | 0.190 | -0.592 | .554 |  |  |
| Technology | 1 | | 0.861 | | 0.704 | 1.222 | .222 |  |  |
| Psychotherapy | 34 | | 0.202 | | 0.099 | 2.042 | .041 |  |  |
| % men_inter_group | 46 | | -0.023 | | 0.014 | -1.713 | .087 |  |  |
|  |  | |  | |  |  |  |  |  |
| **Mean age control group** | | |  | |  |  |  | 0.015 (5) | 0.102 (46) |
| Pharmacotherapy | 11 | | 0.236 | | 0.185 | 1.271 | .204 |  |  |
| Technology | 1 | | 0.318 | | 0.628 | 0.506 | .613 |  |  |
| Psychotherapy | 34 | | 0.045 | | 0.111 | 0.408 | .683 |  |  |
| mean_age_control | 46 | | -0.014 | | 0.013 | -1.060 | .289 |  |  |
|  |  | |  | |  |  |  |  |  |
| **Mean age intervention group** | | |  | |  |  |  | 0.003 (5) | 0.102 (46) |
| Pharmacotherapy | 11 | | 0.259 | | 0.141 | 1.830 | .067 |  |  |
| Technology | 1 | | 0.492 | | 0.633 | 0.777 | .437 |  |  |
| Psychotherapy | 34 | | 0.053 | | 0.086 | 0.615 | .538 |  |  |
| mean_age_intervent | 46 | | -0.015 | | 0.010 | -1.548 | .122 |  |  |
|  |  | |  | |  |  |  |  |  |
| **Intervention: Cognitive training + Social cognition (cont.)** | | | | | | | | | |
|  | *k* | | *g* | | SE | Z | *p*-value |  |  |
| **Education control group** |  | |  | |  |  |  | 0.108 (4) | 0.266 (28) |
| Pharmacotherapy | 11 | | 0.172 | | 0.370 | 0.464 | .643 |  |  |
| Technology | 1 | | 0.349 | | 0.823 | 0.424 | .672 |  |  |
| Psychotherapy | 34 | | -0.020 | | 0.277 | -0.072 | .942 |  |  |
| mean_age_control | 46 | | -0.057 | | 0.121 | -0.471 | .637 |  |  |
|  |  | |  | |  |  |  |  |  |
| **Education intervention group** | | |  | |  |  |  | 0.108 (4) | 0.266 (28) |
| Pharmacotherapy | 11 | | 0.147 | | 0.368 | 0.399 | .690 |  |  |
| Technology | 1 | | 0.387 | | 0.843 | 0.459 | .646 |  |  |
| Psychotherapy | 16 | | -0.025 | | 0.277 | -0.088 | .930 |  |  |
| mean_age_intervent | 28 | | -0.054 | | 0.115 | -0.471 | .637 |  |  |
|  |  | |  | |  |  |  |  |  |
| **Type of medication** |  | |  | |  |  |  | .001 (3) | 0.000 (36) |
| Pharmacotherapy | 11 | | 0.239 | | 0.151 | 1.585 | .113 |  |  |
| Psychotherapy | 25 | | 0.204 | | 0.082 | 2.488 | .013 |  |  |
| Typical antipsychotic | 18 | | -0.088 | | 0.136 | -0.649 | .517 |  |  |
| *Reference category “Atypical antipsychotic” (k=36)* | | | | | | | | |  |
|  |  | |  | |  |  |  |  |  |
| **Amount of medication** | - | |  | |  |  |  |  |  |
|  |  | |  | |  |  |  |  |  |
| **Duration of intervention** |  | |  | |  |  |  | 0.001 (4) | 0.000 (37) |
| Pharmacotherapy | 11 | | 0.194 | | 0.093 | 2.081 | .037 |  |  |
| Technology | 1 | | 0.315 | | 0.530 | 0.593 | .553 |  |  |
| Psychotherapy | 25 | | 0.159 | | 0.068 | 2.319 | .020 |  |  |
| Duration intervention | 37 | | 0.001 | | 0.002 | 0.649 | .517 |  |  |
|  |  | |  | |  |  |  |  |  |
| **Frequency of the intervention** | | |  | |  |  |  | 0.000 (5) | 0.091 (46) |
| Pharmacotherapy | 11 | | 0.268 | | 0.122 | 2.193 | .028 |  |  |
| Technology | 1 | | 0.480 | | 0.614 | 0.782 | .434 |  |  |
| Psychotherapy | 34 | | 0.046 | | 0.079 | 0.579 | .562 |  |  |
| Frequency | 46 | | -0.047 | | 0.023 | -2.028 | .043 |  |  |
|  |  | |  | |  |  |  |  |  |
| **Diagnosis** | - | |  | |  |  |  |  |  |
|  |  | |  | |  |  |  |  |  |
| **Contact control group** |  | |  | |  |  |  | 0.063 (5) | 0.100 (46) |
| Pharmacotherapy | 11 | | 0.151 | | 0.274 | 0.551 | .582 |  |  |
| Technology | 1 | | 0.119 | | 0.716 | 0.166 | .868 |  |  |
| Psychotherapy | 34 | | -0.315 | | 0.402 | -0.782 | .434 |  |  |
| Others | 7 | | 0.279 | | 0.418 | 0.669 | .504 |  |  |
| Therapists | 18 | | 0.371 | | 0.401 | 0.925 | .355 |  |  |
| *Reference category: No contact (k=21). When the reference category was shifted to “Others”, no differences were found neither.* | | | | | | | | | |
|  |  | |  | |  |  |  |  |  |
| **Contact intervention group** | | |  | |  |  |  | 0.063 (5) | 0.100 (46) |
| Pharmacotherapy | 11 | | 0.151 | | 0.274 | 0.551 | .582 |  |  |
| Technology | 1 | | 0.119 | | 0.716 | 0.166 | .868 |  |  |
| Psychotherapy | 34 | | -0.315 | | 0.402 | -0.782 | .434 |  |  |
| **Intervention: Cognitive training + Social cognition (cont.)** | | | | | | | | | |
|  | *k* | | *g* | | SE | Z | *p*-value |  |  |
| Others | 7 | | 0.279 | | 0.418 | 0.669 | .504 |  |  |
| Therapists | 18 | | 0.371 | | 0.401 | 0.925 | .355 |  |  |
| *Reference category: No contact (k=21). When the reference category was shifted to “Others”, no differences were found neither.* | | | | | | | | | |
|  |  | |  | |  |  |  |  |  |
| **Setting** |  | |  | |  |  |  | 0.000 (5) | 0.093 (46) |
| Pharmacotherapy | 11 | | 0.151 | | 0.109 | 1.390 | .165 |  |  |
| Technology | 1 | | 0.270 | | 0.607 | 0.444 | .657 |  |  |
| Psychotherapy | 34 | | 0.179 | | 0.087 | 2.064 | .039 |  |  |
| **Clinic/Home** | 8 | | -0.343 | | 0.176 | -1.956 | .050 |  |  |
| *Reference category: Clinic (k = 37). There are no studies whose setting is at home.* | | | | | | | | | |
|  |  | |  | |  |  |  |  |  |
| **eTable 28. Results of the moderator analyses for Neurocognition. Subset of studies where the TBI intervention was Cognitive training_TBI + CBT_TBI + Vocational therapy_TBI.** | | | | | | | | | |
| **Intervention: Cognitive training + CBT + Vocational therapy**  ***Only data for Psychotherapy control group is available*** | | | | | | | | | |
|  | *k* | | *g* | | SE | Z | *p*-value |  |  |
| **% men in control group** |  | |  | |  |  |  | 0.000 (3) | 0.000 (32) |
| % men_control_group | 32 | | -0.002 | | 0.004 | -0.401 | .688 |  |  |
|  |  | |  | |  |  |  |  |  |
| **% men in intervention group** |  | |  | |  |  |  | 0.000 (3) | 0.000 (32) |
| % men_inter_group | 32 | | -0.002 | | 0.004 | -0.396 | .692 |  |  |
|  |  | |  | |  |  |  |  |  |
| **Mean age control group** |  | |  | |  |  |  | 0.000 (3) | 0.000 (32) |
| Mean age control | 32 | | 0.003 | | 0.007 | 0.422 | .673 |  |  |
|  |  | |  | |  |  |  |  |  |
| **Mean age intervention group** | | |  | |  |  |  | 0.000 (3) | 0.000 (32) |
| Mean age intervent. | 32 | | 0.003 | | 0.006 | 0.441 | .659 |  |  |
|  |  | |  | |  |  |  |  |  |
| **Education control group** |  | |  | |  |  |  | 0.000 (3) | 0.000 (32) |
| Education control | 32 | | -0.012 | | 0.028 | -0.441 | .659 |  |  |
| **Education intervention group** | | |  | |  |  |  | 0.000 (3) | 0.000 (32) |
| Education intervention | 32 | | -0.018 | | 0.041 | -0.435 | .663 |  |  |
|  |  | |  | |  |  |  |  |  |
| **Type of medication** |  | |  | |  |  |  | 0.000 (3) | 0.000 (32) |
| Atypical antipsychotic | 22 | | -0.045 | | 0.147 | -0.308 | .758 |  |  |
| *Reference category: Antipsychotic (k=10)* | | | | |  |  |  |  |  |
|  | | | | |  |  |  |  |  |
| **Amount of medication control** | | | - | |  |  |  |  |  |
|  |  | |  | |  |  |  |  |  |
| **Amount of medication intervention** | | | - | |  |  |  |  |  |
|  |  | |  | |  |  |  |  |  |
| **Intervention: Cognitive training + CBT + Vocational therapy**  ***Only data for Psychotherapy control group is available*** | | | | | | | | | |
|  | *k* | | *g* | | SE | Z | *p*-value |  |  |
| **Duration of intervention** |  | |  | |  |  |  | 0.000 (3) | 0.000 (32) |
| Duration intervention | 32 | | 0.001 | | 0.003 | 0.260 | .795 |  |  |
|  |  | |  | |  |  |  |  |  |
| **Frequency of the intervention** | | |  | |  |  |  |  |  |
| Frequency | 32 | | 0.004 | | 0.033 | 0.120 | .904 | 0.009 (3) | 0.000 (32) |
| **Diagnosis** | - | | - | | - | - | - | - | - |
|  |  | |  | |  |  |  |  |  |
| **Contact control group** |  | |  | |  |  |  | 0.025 (2) | 0.013 (21) |
| Others | 11 | | 0.087 | | 0.180 | 0.485 | .628 |  |  |
| Therapists | 10 | | 0.148 | | 0.211 | 0.701 | .484 |  |  |
| *No difference between categories* | | | | |  |  |  |  |  |
|  | | | | |  |  |  |  |  |
| **Contact intervention group** | | |  | |  |  |  | 0.025 (2) | 0.013 (21) |
| Others | 11 | | 0.087 | | 0.180 | 0.485 | .628 |  |  |
| Therapists | 10 | | 0.148 | | 0.211 | 0.701 | .484 |  |  |
| *No difference between categories* | | | | |  |  |  |  |  |
|  | |  | |  |  |  |  |  |  |
| **Setting** | |  | |  |  |  |  | 0.000 (3) | 0.000 (32) |
| Clinic | | 32 | | -0.014 | 0.007 | 1.931 | .054 |  |  |
| Home | | 0 | | - | - | - | - |  |  |
| Clinic/Home | | 0 | | - | - | - | - |  |  |
| *Notes*. k= number of effect sizes within each category; *g* = overall effect size; SE= standard error; = between-studies variance. In this column, the number of studies analyzed is indicated in brackets; = between-outcomes variance. In this column, the number of effect sizes analyzed is indicated in brackets. | | | | | | | | | |

| **eTable 29. Results of the moderator analyses for Symptomatology. Subset of studies where the TBI intervention was Cognitive training_TBI.** | | | | | | | |
| --- | --- | --- | --- | --- | --- | --- | --- |
| **SYMPTOMATOLOGY** | | | | | | | |
| **Intervention: Cognitive training** | | | | | | | |
|  | *k* | *g* | SE | Z | *p*-value |  |  |
| **% men in control group** |  |  |  |  |  | 0.000 (12) | 0.000 (28) |
| Pharmacotherapy | 8 | 0.058 | 0.133 | 0.435 | .663 |  |  |
| Technology | 8 | -0.154 | 0.130 | -1.178 | .239 |  |  |
| Psychotherapy | 12 | 0.166 | 0.099 | 1.681 | .093 |  |  |
| % men_control_group |  | -0.002 | 0.004 | -0.654 | .513 |  |  |
|  |  |  |  |  |  |  |  |
| **% men in intervention group** |  |  |  |  |  | 0.000 (12) | 0.000 (28) |
| Pharmacotherapy | 8 | 0.070 | 0.132 | 0.534 | .594 |  |  |
| Technology | 8 | -0.155 | 0.129 | -1.203 | .229 |  |  |
| Psychotherapy | 12 | 0.168 | 0.098 | 1.708 | .088 |  |  |
| % men_inter_group |  | -0.003 | 0.004 | -0.744 | .457 |  |  |
|  |  |  |  |  |  |  |  |
| **Mean age control group** |  |  |  |  |  | 0.000 (12) | 0.000 (28) |
| Pharmacotherapy | 8 | 0.081 | 0.133 | 0.606 | .544 |  |  |
| Technology | 8 | -0.208 | 0.137 | -1.515 | .130 |  |  |
| Psychotherapy | 12 | 0.200 | 0.107 | 1.866 | .062 |  |  |
| mean_age_control |  | -0.006 | 0.009 | -0.613 | .540 |  |  |
|  |  |  |  |  |  |  |  |
| **Mean age intervention group** | |  |  |  |  | 0.000 (12) | 0.000 (28) |
| Pharmacotherapy | 8 | 0.089 | 0.136 | 0.650 | .516 |  |  |
| Technology | 8 | -0.209 | 0.141 | -1.489 | .136 |  |  |
| Psychotherapy | 12 | 0.197 | 0.106 | 1.847 | .065 |  |  |
| mean_age_intervent |  | -0.005 | 0.010 | -0.558 | .577 |  |  |
|  |  |  |  |  |  |  |  |
| **Education control group** |  |  |  |  |  | 0.000 (9) | 0.000 (19) |
| Pharmacotherapy | 5 | 0.061 | 0.178 | 0.342 | .732 |  |  |
| Technology | 8 | -0.172 | 0.126 | -1.356 | .175 |  |  |
| Psychotherapy | 6 | 0.082 | 0.148 | 0.551 | .582 |  |  |
| mean_age_control |  | 0.022 | 0.053 | 0.408 | .683 |  |  |
|  |  |  |  |  |  |  |  |
| **Education intervention group** | |  |  |  |  | 0.000 (9) | 0.000 (19) |
| Pharmacotherapy | 5 | 0.126 | 0.250 | 0.505 | .613 |  |  |
| Technology | 8 | -0.185 | 0.159 | -1.163 | .245 |  |  |
| Psychotherapy | 6 | 0.106 | 0.206 | 0.513 | .608 |  |  |
| mean_age_intervent |  | 0.018 | 0.059 | 0.301 | .763 |  |  |
|  |  |  |  |  |  |  |  |
| **Intervention: Cognitive training (cont.)** | | | | | | | |
|  | *k* | *g* | SE | Z | *p*-value |  |  |
| **Type of medication** |  |  |  |  |  | 0.000 (12) | 0.000 (33) |
| Pharmacotherapy | 8 | 0.140 | 0.157 | 0.892 | .373 |  |  |
| Technology | 7 | -0.379 | 0.246 | -1.538 | .124 |  |  |
| Psychotherapy | 18 | 0.207 | 0.169 | 1.227 | .220 |  |  |
| Atypical antipsychotic | 16 | 0.225 | 0.210 | 1.074 | .283 |  |  |
| Typical antipsychotic | 8 | -0.150 | 0.181 | -0.830 | .407 |  |  |
| *Reference category: Antipsychotics (K=9). When the reference category was shifted to “Typical Antipsychotic”, significant differences were found between “Typical Antipsychotic” and “Atypical antipsychotic” (B=0.375, SE=0.180, p-value <.05)* | | | | | | | |
| **Amount of medication control** |  |  |  |  |  | 0.122 (4) | 0.000 (11) |
| Pharmacotherapy | 5 | 0.241 | 0.305 | 0.788 | .431 |  |  |
| Technology | - | - | - | - | - |  |  |
| Psychotherapy | 6 | 0.190 | 0.265 | 0.717 | .474 |  |  |
| Amount medication control |  | 0.002 | 0.001 | 1.133 | .257 |  |  |
|  |  |  |  |  |  |  |  |
| **Amount of medication interven.** | |  |  |  |  | 0.122 (4) | 0.000 (11) |
| Pharmacotherapy | 5 | 0.335 | 0.335 | 1.004 | .316 |  |  |
| Technology | - | - | - | - | - |  |  |
| Psychotherapy | 6 | 0.110 | 0.281 | 0.394 | .694 |  |  |
| Amount medication inter. |  | 0.002 | 0.002 | 1.056 | .291 |  |  |
|  |  |  |  |  |  |  |  |
| **Duration of intervention** |  |  |  |  |  | 0.018 (14) | 0.000 (35) |
| Pharmacotherapy | 8 | 0.098 | 0.160 | 0.610 | .542 |  |  |
| Technology | 8 | -0.184 | 0.143 | -1.290 | .197 |  |  |
| Psychotherapy | 19 | 0.227 | 0.103 | 2.217 | .027 |  |  |
| Duration intervention |  | 0.004 | 0.030 | 0.116 | .908 |  |  |
|  |  |  |  |  |  |  |  |
| **Frequency** |  |  |  |  |  | 0.015 (14) | 0.000 (35) |
| Pharmacotherapy | 8 | 0.116 | 0.161 | 0.720 | .471 |  |  |
| Technology | 8 | -0.212 | 0.152 | -1.394 | .163 |  |  |
| Psychotherapy | 19 | 0.226 | 0.099 | 2.282 | .023 |  |  |
| Frequency |  | 0.016 | 0.031 | 0.505 | .613 |  |  |
|  |  |  |  |  |  |  |  |
| **Diagnosis** |  |  |  |  |  | 0.013 (14) | 0.000 (35) |
| Pharmacotherapy | 8 | 0.088 | 0.150 | 0.585 | .559 |  |  |
| Technology | 8 | -0.091 | 0.167 | -0.546 | .585 |  |  |
| Psychotherapy | 19 | 0.241 | 0.098 | 2.450 | .014 |  |  |
| Diagnosis (Schizophrenia) | 29 | -0.239 | 0.247 | -0.969 | .333 |  |  |
| *Reference category Psychosis (k=6)* | |  |  |  |  |  |  |
|  |  |  |  |  |  |  |  |
| **Contact control group** |  |  |  |  |  |  |  |
| Pharmacotherapy | 8 | 0.069 | 0.132 | 0.526 | .599 |  |  |
| Technology | 8 | -0.136 | 0.129 | -1.057 | .291 |  |  |
| Psychotherapy | 19 | 0.233 | 0.109 | 2.126 | .034 |  |  |
| Others | 4 | -0.284 | 0.183 | -1.548 | .122 |  |  |
| Therapists | 6 | 0.287 | 0.203 | 1.410 | .159 |  |  |
| *Reference category: No contact (k=25). When the reference category was shifted to “Others”, significant differences were found between “Others” and “Therapist” (B = 0.510, SE=0.236, p < .05).* | | | | | | | |
| **Intervention: Cognitive training (cont.)** | | | | | | | |
|  | *k* | *g* | SE | Z | *p*-value |  |  |
| **Contact intervention group** | |  |  |  |  | 1 | 35 |
| Pharmacotherapy | 8 | 0.066 | 0.133 | 0.494 | .621 |  |  |
| Technology | 7 | -0.179 | 0.129 | -1.394 | .163 |  |  |
| Psychotherapy | 18 | 0.126 | 0.171 | 0.736 | .462 |  |  |
| Others | 13 | 0.032 | 0.179 | 0.177 | .860 |  |  |
| Therapists | 6 | 0.394 | 0.242 | 1.626 | .104 |  |  |
| **Setting** |  |  |  |  |  | 0.013 (14) | 0.000 (35) |
| Pharmacotherapy | 8 | 0.166 | 0.199 | 0.835 | .404 |  |  |
| Technology | 7 | -0.078 | 0.219 | -0.358 | .721 |  |  |
| Psychotherapy | 18 | 0.364 | 0.243 | 1.498 | .134 |  |  |
| Clinic | 31 | -0.134 | 0.223 | -0.600 | .549 |  |  |
| *Reference category: Home (k = 4). There are no studies whose setting is at clinic and home.* | | | | | | | |
| **eTable 30. Results of the moderator analyses for Symptomatology. Subset of studies where the TBI intervention was Cognitive training_TBI + Social cognition_TBI.** | | | | | | | |
| **Intervention: Cognitive training + Social cognition** | | | | | | | |
|  | *k* | *g* | SE | Z | *p*-value |  |  |
| **% men in control group** |  |  |  |  |  | 0.000 (4) | 2.351 (20) |
| Pharmacotherapy | 3 | 0.008 | 1.499 | 0.005 | .996 |  |  |
| Technology | - | - | - | - | - |  |  |
| Psychotherapy | 17 | 0.361 | 0.361 | 0.830 | .406 |  |  |
| % men_control_group |  | -0.017 | 0.091 | -0.182 | .856 |  |  |
|  |  |  |  |  |  |  |  |
| **% men in intervention group** |  |  |  |  |  | 0.000 (4) | 2.354 (20) |
| Pharmacotherapy | 3 | 0.314 | 1.283 | 0.245 | .806 |  |  |
| Technology | - | - | - | - | - |  |  |
| Psychotherapy | 17 | 0.307 | 0.415 | 0.741 | .459 |  |  |
| % men_inter_group |  | 0.006 | 0.064 | 0.095 | .924 |  |  |
|  |  |  |  |  |  |  |  |
| **Mean age control group** |  |  |  |  |  | 0.000 (4) | 2.356 (20) |
| Pharmacotherapy | 3 | 0.222 | 0.960 | 0.231 | .817 |  |  |
| Technology | - | - | - | - | - |  |  |
| Psychotherapy | 17 | 0.324 | 0.386 | 0.840 | .401 |  |  |
| mean_age_control |  | 0.001 | 0.046 | 0.014 | .989 |  |  |
|  |  |  |  |  |  |  |  |
| **Mean age intervention group** |  |  |  |  |  | 0.000 (4) | 2.354 (20) |
| Pharmacotherapy | 3 | 0.202 | 0.952 | 0.212 | .832 |  |  |
| Technology | - | - | - | - | - |  |  |
| Psychotherapy | 17 | 0.327 | 0.385 | 0.850 | .395 |  |  |
| mean_age_intervent |  | 0.003 | 0.044 | 0.075 | .940 |  |  |
|  |  |  |  |  |  |  |  |
| **Education control group** |  |  |  |  |  | 0.000 (3) | 3.298 (12) |
| Pharmacotherapy | 3 | 0.252 | 1.057 | 0.238 | .812 |  |  |
| Technology | - | - | - | - | - |  |  |
| Psychotherapy | 9 | 0.326 | 0.615 | 0.530 | .596 |  |  |
| Education control |  | 0.109 | 0.284 | 0.385 | .701 |  |  |
|  |  |  |  |  |  |  |  |
| **Intervention: Cognitive training + Social cognition (cont.)** | | | | | | | |
|  | *k* | *g* | SE | Z | *p*-value |  |  |
| **Education intervention group** |  |  |  |  |  | 0.934 (3) | 4.456 (12) |
| Pharmacotherapy | 3 | 0.282 | 1.575 | 0.179 | .858 |  |  |
| Technology | - | - | - | - | - |  |  |
| Psychotherapy | 9 | 0.318 | 1.004 | 0.317 | .751 |  |  |
| Education intervention |  | 0.104 | 0.422 | 0.247 | .805 |  |  |
| **Type of medication** |  |  |  |  |  | 0.001 (3) | 0.000 (14) |
| Pharmacotherapy | 3 | 0.278 | 0.127 | 2.186 | .029 |  |  |
| Technology | - | - | - | - | - |  |  |
| Psychotherapy | 11 | 0.227 | 0.116 | 1.952 | .051 |  |  |
| Atypical antipsychotic | 8 | -0.293 | 0.165 | -0.090 | .928 |  |  |
| *Reference category “Typical antipsychotic” (k=6)* | | | | | |  |  |
|  |  |  |  |  |  |  |  |
| **Amount of medication control** | | - |  |  |  |  |  |
| **Amount of medication intervention** | | - |  |  |  |  |  |
|  |  |  |  |  |  |  |  |
| **Duration of intervention** |  |  |  |  |  | 0.001 (3) | 0.000 (14) |
| Pharmacotherapy | 3 | 0.394 | 0.166 | 2.372 | .018 |  |  |
| Technology | - | - | - | - | - |  |  |
| Psychotherapy | 11 | 0.153 | 0.101 | 1.506 | .132 |  |  |
| Duration intervention |  | 0.004 | 0.003 | 1.409 | .159 |  |  |
|  |  |  |  |  |  |  |  |
| **Frequency** |  |  |  |  |  | 0.000 (3) | 2.346 (20) |
| Pharmacotherapy | 3 | 0.158 | 0.926 | 0.171 | .865 |  |  |
| Technology | - | - | - | - | - |  |  |
| Psychotherapy | 17 | 0.335 | 0.383 | 0.875 | .382 |  |  |
| Frequency |  | 0.029 | 0.105 | 0.273 | .785 |  |  |
|  |  |  |  |  |  |  |  |
| **Diagnosis** |  |  |  |  |  |  |  |
| *All schizophrenia* |  |  |  |  |  |  |  |
|  | | | |  |  |  |  |
| **Contact control group** |  |  |  |  |  |  |  |
| **-** |  |  |  |  |  |  |  |
| **Contact intervention group** | |  |  |  |  |  |  |
| **-** |  |  |  |  |  |  |  |
|  |  |  |  |  |  |  |  |
| **Setting** |  |  |  |  |  | 0.013 (14) | 0.000 (35) |
| Pharmacotherapy | 3 | 0.496 | 1.193 | 0.415 | .678 |  |  |
| Technology | - | - | - | - | - |  |  |
| Psychotherapy | 17 | 0.497 | 0.639 | 0.778 | .437 |  |  |
| Clinic | 14 | -0.269 | 0.794 | -0.339 | .735 |  |  |
| *Reference category: Clinic/Home (k = 6). There are no studies whose setting is at home.* | | | | | | | |

*Notes*. k = number of effect sizes within each category; *g* = overall effect size; SE= standard error; = between-studies variance. In this column, the number of studies analyzed is indicated in brackets; = between-outcomes variance. In this column, the number of effect sizes analyzed is indicated in brackets.

| **eTable 31. Results of the moderator analyses for Functioning. Subset of studies where the TBI intervention was Cognitive training_TBI.** | | | | | | | | | | |  |
| --- | --- | --- | --- | --- | --- | --- | --- | --- | --- | --- | --- |
| **FUNCTIONING** | | | | | | | | | | |  |
| **Intervention: Cognitive training** | | | | | | | | | | |  |
|  | | *k* | *g* | SE | | Z | *p*-value |  |  | |  |
| **% men in control group** | |  |  |  | |  |  | 0.000 (10) | 0.381 (20) | |  |
| Pharmacotherapy | | 3 | 0.058 | 0.459 | | 0.127 | .899 |  |  | |  |
| Technology | | 8 | -0.151 | 0.292 | | -0.517 | .605 |  |  | |  |
| Psychotherapy | | 9 | 0.319 | 0.259 | | 1.232 | .218 |  |  | |  |
| % men_control_group | |  | 0.012 | 0.013 | | 0.904 | .366 |  |  | |  |
|  | |  |  |  | |  |  |  |  | |  |
| **% men in intervention group** | |  |  |  | |  |  | 0.000 (10) | 0.354 (20) | |  |
| Pharmacotherapy | | 3 | 0.053 | 0.434 | | 0.122 | .903 |  |  | |  |
| Technology | | 8 | -0.263 | 0.306 | | -0.861 | .389 |  |  | |  |
| Psychotherapy | | 9 | 0.400 | 0.267 | | 1.500 | .134 |  |  | |  |
| % men_inter_group | |  | 0.018 | 0.014 | | 1.340 | .180 |  |  | |  |
|  | |  |  |  | |  |  |  |  | |  |
| **Mean age control group** | |  |  |  | |  |  | 0.010 (10) | 0.407 (20) | |  |
| Pharmacotherapy | | 3 | -0.093 | 0.451 | | -0.206 | .837 |  |  | |  |
| Technology | | 8 | 0.027 | 0.278 | | 0.097 | .923 |  |  | |  |
| Psychotherapy | | 9 | 0.190 | 0.265 | | 0.718 | .473 |  |  | |  |
| mean_age_control | |  | 0.008 | 0.017 | | 0.432 | .666 |  |  | |  |
|  | |  |  |  | |  |  |  |  | |  |
| **Mean age intervention group** | | |  |  | |  |  | 0.012 (10) | 0.406 (20) | |  |
| Pharmacotherapy | | 3 | -0.091 | 0.451 | | -0.201 | .841 |  |  | |  |
| Technology | | 8 | 0.027 | 0.281 | | 0.096 | .924 |  |  | |  |
| Psychotherapy | | 9 | 0.187 | 0.268 | | 0.700 | .484 |  |  | |  |
| mean_age_intervent | |  | 0.007 | 0.018 | | 0.407 | .684 |  |  | |  |
|  | |  |  |  | |  |  |  |  | |  |
| **Education control group** | |  |  |  | |  |  | 0.000 (8) | 0.274 (17) | |  |
| Pharmacotherapy | | 2 | -0.012 | 0.506 | | -0.023 | .982 |  |  | |  |
| Technology | | 8 | -0.046 | 0.226 | | -0.203 | .839 |  |  | |  |
| Psychotherapy | | 7 | 0.466 | 0.249 | | 1.868 | .062 |  |  | |  |
| mean_age_control | |  | -0.073 | 0.111 | | -0.658 | .511 |  |  | |  |
|  | |  |  |  | |  |  |  |  | |  |
| **Education intervention group** | | |  |  | |  |  | 0.000 (8) | 0.447 (17) | |  |
| Pharmacotherapy | | 2 | -0.005 | 0.603 | | -0.008 | .993 |  |  | |  |
| Technology | | 8 | -0.038 | 0.273 | | -0.139 | .890 |  |  | |  |
| Psychotherapy | | 7 | 0.476 | 0.296 | | 1.607 | .108 |  |  | |  |
| mean_age_intervent | |  | -0.049 | 0.122 | | -0.397 | .691 |  |  | |  |
|  | |  |  |  | |  |  |  |  | |  |
| **Type of medication** | |  |  |  | |  |  |  |  | |  |
| Pharmacotherapy | | 3 | -0.077 | 0.411 | | -0.187 | .852 |  |  | |  |
| Technology | | 8 | 0.097 | 0.961 | | 0.101 | .920 |  |  | |  |
| Psychotherapy | | 9 | -0.356 | 0.810 | | -0.440 | .660 |  |  | |  |
| Atypical antipsychotic | | 10 | -0.111 | 0.932 | | -0.120 | .905 |  |  | |  |
| Typical antipsychotic | | 6 | 0.894 | 0.855 | | 1.045 | .296 |  |  | |  |
| *Reference category: Antipsychotics (k=4). When the reference category was shifted to “Typical Antipsychotic”, no differences were found neither.* | | | | | | | | | | |  |
| **Intervention: Cognitive training (cont.)** | | | | | | | | | | |  |
| *k* | | | *g* | SE | | Z | *p*-value |  |  | |  |
| **Amount of medication control** | | |  |  | |  |  | 0.240 (3) | 1.126 (9) | |  |
| Pharmacotherapy | | 2 | 0.409 | 1.074 | | 0.381 | .703 |  |  | |  |
| Technology | | - | - | - | | - | - |  |  | |  |
| Psychotherapy | | 7 | 0.210 | 0.560 | | 0.375 | .708 |  |  | |  |
| Amount medication control | |  | -0.003 | 0.003 | | -0.985 | .325 |  |  | |  |
|  | |  |  |  | |  |  |  |  | |  |
| **Amount of medication intervention** | | |  |  | |  |  | 0.240 (3) | 1.126 (9) | |  |
| Pharmacotherapy | | 2 | 0.110 | 0.974 | | 0.113 | .910 |  |  | |  |
| Technology | | - | - | - | | - | - |  |  | |  |
| Psychotherapy | | 7 | 0.296 | 0.560 | | 0.528 | .598 |  |  | |  |
| Amount medication inter. | |  | -0.003 | 0.003 | | -0.985 | .325 |  |  | |  |
|  | |  |  |  | |  |  |  |  | |  |
| **Duration of intervention** | |  |  |  | |  |  | 0.003 (11) | 0.383 (21) | |  |
| Pharmacotherapy | | 3 | -0.075 | 0.437 | | -0.173 | .863 |  |  | |  |
| Technology | | 8 | -0.010 | 0.257 | | -0.038 | .970 |  |  | |  |
| Psychotherapy | | 10 | 0.238 | 0.237 | | 1.003 | .316 |  |  | |  |
| Duration intervention | |  | 0.006 | 0.069 | | 0.080 | .937 |  |  | |  |
|  | |  |  |  | |  |  |  |  | |  |
| **Frequency** | |  |  |  | |  |  | 0.013 (11) | 0.375 (21) | |  |
| Pharmacotherapy | | 3 | -0.062 | 0.466 | | -0.132 | .895 |  |  | |  |
| Technology | | 8 | -0.024 | 0.273 | | -0.088 | .930 |  |  | |  |
| Psychotherapy | | 10 | 0.232 | 0.238 | | 0.974 | .330 |  |  | |  |
| Frequency | |  | 0.007 | 0.068 | | 0.098 | .922 |  |  | |  |
|  | |  |  |  | |  |  |  |  | |  |
| **Diagnosis** | |  |  |  | |  |  | 0.010 (11) | 0.367 (21) | |  |
| Pharmacotherapy | | 3 | -0.077 | 0.434 | | -0.177 | .860 |  |  | |  |
| Technology only | | 8 | -0.060 | 0.273 | | -0.219 | .827 |  |  | |  |
| Psychotherapy | | 10 | 0.212 | 0.238 | | 0.893 | .372 |  |  | |  |
| Diagnosis (Schizophrenia) | | 18 | 0.210 | 0.487 | | 0.431 | .666 |  |  | |  |
| *Reference category Psychosis (k=3)* | | |  |  | |  |  |  |  | |  |
|  | |  |  |  | |  |  |  |  | |  |
| **Contact control group** | |  |  |  | |  |  | 0.000 (11) | 0.371 (21) | |  |
| Pharmacotherapy | | 3 | 0.198 | 0.583 | | 0.339 | .735 |  |  | |  |
| Technology | | 8 | 0.227 | 0.425 | | 0.534 | .593 |  |  | |  |
| Psychotherapy | | 10 | 0.354 | 0.278 | | 1.276 | .202 |  |  | |  |
| Others | | 7 | 0.275 | 0.395 | | 0.696 | .486 |  |  | |  |
| *Reference category: No contact (k=14)* | | | | | | | | | | |  |
|  | |  |  |  | |  |  |  |  | |  |
| **Contact intervention group** | | |  |  | |  |  | 11 (0.060) | 21 | |  |
| Pharmacotherapy | | 3 | -0.241 | 0.324 | | -0.742 | .458 |  |  | |  |
| Technology | | 8 | -0.317 | 0.289 | | -1.098 | .272 |  |  | |  |
| Psychotherapy | | 10 | -0.025 | 0.190 | | -0.131 | .896 |  |  | |  |
| Others | | 11 | -0.338 | 0.273 | | -1.238 | .216 |  |  | |  |
| *Reference category: No contact (k=10).*  *In this analysis, a standard random-effects model was fitted because the three-level model did not converge.* | | | | | | | | | | |  |
| **Intervention: Cognitive training (cont.)** | | | | | | | | | | |  |
|  | | *k* | *g* | SE | | Z | *p*-value |  |  | |  |
| **Setting** | |  |  |  | |  |  | 0.013 (11) | 0.377 (21) | |  |
| Pharmacotherapy | | 3 | -0.109 | 0.522 | | -0.210 | .834 |  |  | |  |
| Technology | | 8 | -0.046 | 0.371 | | -0.123 | .902 |  |  | |  |
| Psychotherapy | | 10 | 0.177 | 0.511 | | 0.347 | .729 |  |  | |  |
| Clinic | | 17 | 0.052 | 0.452 | | 0.116 | .116 |  |  | |  |
| *Reference category: Home (k = 4). There are no studies whose setting is at clinic and home.* | | | | | | | | | | | |
| **eTable 32. Results of the moderator analyses for Functioning. Subset of studies where the TBI intervention was Cognitive training_TBI + Social cognition_TBI.** | | | | | | | | | | |  |
| **Intervention: Cognitive training + Social cognition** | | | | | | | | | | |  |
|  | | *k* | *g* | SE | | Z | *p*-value |  |  | |  |
| **% men in control group** | |  |  |  | |  |  | 0.000 (4) | 0.937 (36) | |  |
| Pharmacotherapy | | 2 | 1.618 | 1.246 | | 1.298 | .194 |  |  | |  |
| Technology | | - | - | - | | - | - |  |  | |  |
| Psychotherapy | | 34 | 0.642 | 0.189 | | 3.393 | .0007 |  |  | |  |
| % men_control_group | |  | 0.096 | 0.060 | | 1.589 | .112 |  |  | |  |
|  | |  |  |  | |  |  |  |  | |  |
| **% men in intervention group** | |  |  |  | |  |  | 0.075 (4) | 0.950 (36) | |  |
| Pharmacotherapy | | 2 | -0.728 | 0.969 | | -0.751 | .452 |  |  | |  |
| Technology | | - | - | - | | - | - |  |  | |  |
| Psychotherapy | | 34 | 0.790 | 0.294 | | 2.684 | .007 |  |  | |  |
| % men_inter_group | |  | -0.057 | 0.049 | | -1.159 | .247 |  |  | |  |
|  | |  |  |  | |  |  |  |  | |  |
| **Mean age control group** | |  |  |  | |  |  | 0.227 (4) | 0.956 (36) | |  |
| Pharmacotherapy | | 2 | 0.407 | 1.022 | | 0.399 | .690 |  |  | |  |
| Technology | | - | - | - | | - | - |  |  | |  |
| Psychotherapy | | 34 | 0.697 | 0.399 | | 1.746 | .081 |  |  | |  |
| mean_age_control | |  | -0.033 | 0.044 | | -0.757 | .449 |  |  | |  |
|  | |  |  |  | |  |  |  |  | |  |
| **Mean age intervention group** | |  |  |  | |  |  | 0.113 (4) | 0.952 (36) | |  |
| Pharmacotherapy | | 2 | 0.472 | 0.913 | | 0.517 | .605 |  |  | |  |
| Technology | | - | - | - | | - | - |  |  | |  |
| Psychotherapy | | 34 | 0.717 | 0.310 | | 2.314 | .021 |  |  | |  |
| mean_age_intervent | |  | -0.037 | 0.036 | | -1.040 | .298 |  |  | |  |
|  | |  |  |  | |  |  |  |  | |  |
| **Education control group** | |  |  |  | |  |  | 0.000 (3) | 2.714 (10) | |  |
| Pharmacotherapy | | 2 | 0.192 | 1.194 | | 0.161 | .872 |  |  | |  |
| Technology | | - | - | - | | - | - |  |  | |  |
| Psychotherapy | | 8 | 0.361 | 0.600 | | 0.601 | .545 |  |  | |  |
| Education control | |  | -0.260 | 0.269 | | -0.969 | .333 |  |  | |  |
|  | |  |  |  | |  |  |  |  | |  |
| **Intervention: Cognitive training + Social cognition (cont.)** | | | | | | | | | | |  |
|  | | *k* | *g* | SE | | Z | *p*-value |  |  | |  |
| **Education intervention group** | |  |  |  | |  |  | 0.987 (3) | 4.097 (10) | |  |
| Pharmacotherapy | | 2 | 0.122 | 1.764 | | 0.069 | .945 |  |  | |  |
| Technology | | - | - | - | | - | - |  |  | |  |
| Psychotherapy | | 8 | 0.393 | 1.022 | | 0.385 | .700 |  |  | |  |
| Education intervention | |  | -0.257 | 0.427 | | -0.602 | .547 |  |  | |  |
|  | |  |  |  | |  |  |  |  | |  |
| **Type of medication** | |  |  |  | |  |  | 0.224 (3) | 0.516 (33) | |  |
| Pharmacotherapy | | 2 | -0.023 | 0.707 | | -0.032 | .974 |  |  | |  |
| Technology | | - | - | - | | - | - |  |  | |  |
| Psychotherapy | | 31 | 0.622 | 0.594 | | 1.047 | .295 |  |  | |  |
| Atypical antipsychotic | | 26 | 0.192 | 0.777 | | .290 | .772 |  |  | |  |
| *Reference category “Typical antipsychotic” (k=7)* | | | | | | | |  |  | |  |
|  | | |  |  | |  |  |  |  | |  |
| **Amount of medication control** | | | - | - | | - | - |  |  | |  |
|  | | |  |  | |  |  |  |  | |  |
| **Amount of medication intervention** | | | - | - | | - | - |  |  | |  |
|  | |  |  |  | |  |  |  |  | |  |
| **Duration of intervention** | |  |  |  | |  |  | 0.224 (3) | 0.516 (33) | |  |
| Pharmacotherapy | | 2 | 0.155 | 0.935 | | 0.166 | .869 |  |  | |  |
| Technology | | - | - | - | | - | - |  |  | |  |
| Psychotherapy | | 31 | 0.800 | 0.414 | | 1.933 | .053 |  |  | |  |
| Duration intervention | |  | 0.003 | 0.009 | | 0.290 | .772 |  |  | |  |
|  | |  |  |  | |  |  |  |  | |  |
| **Frequency** | |  |  |  | |  |  | 0.000 (4) | 0.931 (36) | |  |
| Pharmacotherapy | | 2 | 0.624 | 0.786 | | 0.794 | .428 |  |  | |  |
| Technology | | - | - | - | | - | - |  |  | |  |
| Psychotherapy | | 34 | 0.700 | 0.181 | | 3.878 | <.001 |  |  | |  |
| Frequency | |  | -0.137 | 0.078 | | -1.767 | .077 |  |  | |  |
|  | |  |  | | | |  |  |  | |  |
| **Diagnosis** | | - | *All schizophrenia* | | | |  |  |  | |  |
|  | |  |  |  | |  |  |  |  | |  |
| **Contact control group** | |  |  |  | |  |  | 0.312 (4) | 0.979 (36) | |  |
| Pharmacotherapy | | 2 | -0.023 | 0.906 | | -0.025 | .980 |  |  | |  |
| Technology | | - | - | - | | - | - |  |  | |  |
| Psychotherapy | | 34 | -0.276 | 0.826 | | -0.334 | .738 |  |  | |  |
| Others | | 5 | 1.012 | 1.104 | | 0.917 | .359 |  |  | |  |
| Therapist | | 26 | 1.133 | 1.019 | | 1.112 | .266 |  |  | |  |
| *Reference category: No contact (k=5). When the reference category was shifted to “Other”, no statistical difference was found neither.* | | | | | | | | | | |  |
|  | | |  |  | |  |  |  |  | |  |
| **Contact intervention group** | | |  |  | |  |  | 0.312 (4) | 0.979 (36) | |  |
| Pharmacotherapy | | 2 | -0.023 | 0.906 | | -0.025 | .980 |  |  | |  |
| Technology | | - | - | - | | - | - |  |  | |  |
| Psychotherapy | | 34 | -0.276 | 0.826 | | -0.334 | .738 |  |  | |  |
| Others | | 5 | 1.133 | 1.019 | | 1.112 | .266 |  |  | |  |
| Therapist | | 25 | 1.012 | 1.104 | | 0.917 | .359 |  |  | |  |
| *Reference category: No contact (k=5)* | | | |  | |  |  |  |  | |  |
|  | | | |  | |  |  |  |  | |  |
| **Intervention: Cognitive training + Social cognition (cont.)** | | | | | | | | | | |  |
|  | | *k* | *g* | SE | | Z | *p*-value |  |  | |  |
| **Setting** | | | |  | |  |  | 0.000 (4) | 0.946 (36) | |  |
| Pharmacotherapy | 2 | | -1.132 | | 0.942 | -1.202 | .229 |  | |  |  |
| Technology | - | | - | | - | - | - |  | |  |  |
| Psychotherapy | 34 | | -0.273 | | 0.599 | -0.456 | .648 |  | |  |  |
| Clinic | 33 | | 1.109 | | 0.628 | 1.766 | .078 |  | |  |  |
| *Reference category: Clinic/Home (k = 3). There are no studies whose setting is at home only.* | | | | | | | | | | |  |

*Notes*. k= number of effect sizes within each category; *g* = overall effect size; SE= standard error; = between-studies variance. In this column, the number of studies analyzed is indicated in brackets; = between-outcomes variance. In this column, the number of effect sizes analyzed is indicated in brackets.

**eTable 33. Results of the moderator analyses for Social cognition. Subset of studies where the TBI intervention was Cognitive training_TBI + Social cognition_TBI.**

| **SOCIAL COGNITION** | | | | | | | | | | |
| --- | --- | --- | --- | --- | --- | --- | --- | --- | --- | --- |
| **Intervention: Cognitive training + Social cognition** | | | | | | | | | | |
|  | *k* | *g* | SE | Z | *p*-value |  | |  | | |
| **% men in control group** |  |  |  |  |  | 0.000 (5) | | 1.085 (27) | | |
| Pharmacotherapy | 1 | -1.300 | 1.473 | -0.882 | .378 |  | |  | | |
| Technology | 1 | 0.583 | 1.174 | 0.497 | .620 |  | |  | | |
| Psychotherapy | 25 | 0.442 | 0.223 | 1.978 | .048 |  | |  | | |
| % men_control_group |  | -0.102 | 0.063 | -1.610 | .107 |  | |  | | |
|  |  |  |  |  |  |  | |  | | |
| **% men in intervention group** |  |  |  |  |  | 0.022 (5) | | 1.110 (27) | | |
| Pharmacotherapy | 1 | 1.253 | 1.282 | 0.978 | .328 |  | |  | | |
| Technology | 1 | -0.862 | 1.515 | -0.569 | .569 |  | |  | | |
| Psychotherapy | 25 | 0.399 | 0.243 | 1.643 | .100 |  | |  | | |
| % men_inter_group |  | 0.058 | 0.044 | 1.314 | .189 |  | |  | | |
|  |  |  |  |  |  |  | |  | | |
| **Mean age control group** |  |  |  |  |  | 0.156 (5) | | 1.110 (27) | | |
| Pharmacotherapy | 1 | 0.015 | 1.205 | 0.012 | .990 |  | |  | | |
| Technology | 1 | 0.131 | 1.274 | 0.103 | .918 |  | |  | | |
| Psychotherapy | 25 | 0.432 | 0.333 | 1.296 | .195 |  | |  | | |
| mean_age_control |  | 0.034 | 0.040 | 0.862 | .389 |  | |  | | |
|  |  |  |  |  |  |  | |  | | |
| **Mean age intervention group** |  |  |  |  |  | 0.055 (5) | | 1.110 (27) | | |
| Pharmacotherapy | 1 | -0.021 | 1.140 | -0.018 | .985 |  | |  | | |
| Technology | 1 | -0.282 | 1.323 | -0.213 | .832 |  | |  | | |
| Psychotherapy | 25 | 0.431 | 0.267 | 1.611 | .107 |  | |  | | |
| mean_age_intervent |  | 0.038 | 0.033 | 1.179 | .239 |  | |  | | |
|  |  |  |  |  |  |  | |  | | |
| **Education control group** |  |  |  |  |  | 0.000 (4) | | 1.328 (13) | | |
| Pharmacotherapy | 1 | 0.186 | 1.176 | 0.158 | .874 |  | |  | | |
| Technology | 1 | 0.011 | 1.303 | 0.008 | .993 |  | |  | | |
| Psychotherapy | 11 | 0.742 | 0.362 | 2.052 | .040 |  | |  | | |
| Education control |  | 0.194 | 0.163 | 1.191 | .234 |  | |  | | |
|  |  |  |  |  |  |  | |  | | |
| **Education intervention group** |  |  |  |  |  | 0.446 (4) | | 1.992 (13) | | |
| Pharmacotherapy | 1 | 0.259 | 1.578 | 0.164 | .870 |  | |  | | |
| Technology | 1 | -0.143 | 1.804 | -0.079 | .937 |  | |  | | |
| Psychotherapy | 11 | 0.760 | 0.649 | 1.172 | .241 |  | |  | | |
| Education intervention |  | 0.191 | 0.272 | 0.703 | .482 |  | |  | | |
|  |  |  |  |  |  |  | |  | | |
| **Type of medication** |  |  |  |  |  | 0.091 (3) | | 0.252 (22) | | |
| Pharmacotherapy | 1 | 0.163 | 0.800 | 0.203 | .839 |  | |  | | |
| Technology | 1 | - | - | - | - |  | |  | | |
| Psychotherapy | 25 | 0.181 | 0.350 | 0.517 | .605 |  | |  | | |
| Atypical antipsychotic | 14 | -0.186 | 0.515 | -0.364 | .716 |  | |  | | |
| *Reference category “Typical antipsychotic” (k=8)* | | | | | |  | |  | | |
|  |  |  |  |  |  |  | |  | | |
| **Intervention: Cognitive training + Social cognition (cont.)** | | | | | | | | | | |
|  | *k* | *g* | SE | Z | *p*-value |  | |  | | |
| **Amount of medication control** | | - |  |  |  |  | |  | | |
| **Amount of medication intervention** | | - |  |  |  |  | |  | | |
|  |  |  |  |  |  |  | |  | | |
| **Duration of intervention** |  |  |  |  |  | 0.085 (4) | | 0.252 (23) | | |
| Pharmacotherapy | 1 | 0.236 | 0.682 | 0.345 | .730 |  | |  | | |
| Technology | 1 | 0.256 | 0.845 | 0.304 | .762 |  | |  | | |
| Psychotherapy | 21 | 0.254 | 0.252 | 1.009 | .313 |  | |  | | |
| Duration intervention |  | -0.002 | 0.006 | -0.373 | .709 |  | |  | | |
|  |  |  |  |  |  |  | |  | | |
| **Frequency** |  |  |  |  |  | 0.000 (5) | | 1.066 (27) | | |
| Pharmacotherapy | 1 | -0.165 | 1.090 | -0.151 | .880 |  | |  | | |
| Technology | 1 | -0.412 | 1.242 | -0.332 | .740 |  | |  | | |
| Psychotherapy | 25 | 0.439 | 0.221 | 1.984 | .047 |  | |  | | |
| Frequency |  | 0.136 | 0.077 | 1.758 | .079 |  | |  | | |
|  |  |  |  |  |  |  | |  | | |
| **Diagnosis** |  |  |  |  |  |  | |  | | |
| *All schizophrenia* |  |  |  |  |  |  | |  | | |
|  | | | |  |  |  | |  | | |
| **Contact control group** |  |  |  |  |  | 0.287 (5) | | 1.124 (27) | | |
| Pharmacotherapy | 1 | 0.349 | 1.203 | 0.290 | .772 |  | |  | | |
| Technology | 1 | 0.374 | 1.299 | 0.288 | .774 |  | |  | | |
| Psychotherapy | 25 | 1.247 | 0.772 | 1.616 | .106 |  | |  | | |
| Others | 7 | -0.877 | 1.027 | -0.854 | .393 |  | |  | | |
| Therapist | 14 | -1.095 | 0.988 | -1.108 | .268 |  | |  | | |
| *Reference category: No contact (k=6). When the reference category was shifted to “Other”, no statistical difference was found neither.* | | | | | | | | | | |
|  |  |  |  |  |  |  | |  | | |
| **Contact intervention group** | |  |  |  |  | 0.287 (5) | | 1.124 (27) | | |
| Pharmacotherapy | 1 | 0.349 | 1.203 | 0.290 | .772 |  | |  | | |
| Technology | 1 | 0.374 | 1.299 | 0.288 | .774 |  | |  | | |
| Psychotherapy | 25 | 1.247 | 0.772 | 1.616 | .106 |  | |  | | |
| Others | 7 | -1.095 | 0.988 | -1.108 | .268 |  | |  | | |
| Therapist | 14 | -0.877 | 1.027 | -0.854 | .393 |  | |  | | |
| *Reference category: No contact (k=6). When the reference category was shifted to “Other”, no statistical difference was found neither.* | | | | | | | | | | |
|  | | | | | | | | | | |
| **Setting** |  |  |  |  |  | | 0.000 (5) | | 1.075 (27) |  |
| Pharmacotherapy | 1 | 1.362 | 1.211 | 1.125 | .261 | |  | |  |  |
| Technology | 1 | 1.387 | 1.306 | 1.062 | .288 | |  | |  |  |
| Psychotherapy | 25 | 1.243 | 0.544 | 2.284 | .022 | |  | |  |  |
| Clinic | 23 | -1.013 | 0.595 | -1.702 | .089 | |  | |  |  |
| *Reference category: Clinic/Home (k = 4). There are no studies whose setting is at home only.* | | | | | | | | | |  |

*Notes*. k= number of effect sizes within each category; *g* = overall effect size; SE= standard error; = between-studies variance. In this column, the number of studies analyzed is indicated in brackets; = between-outcomes variance. In this column, the number of effect sizes analyzed is indicated in brackets.

**eAppendix 7. Publication bias analyses**

**eFigure 31. Funnel plot for effect sizes of Neurocognition domain**

**eFigure 32. Funnel plot for effect sizes of Symptomatology domain**

**eFigure 33. Funnel plot for effect sizes of Functioning domain**

**eFigure 34. Funnel plot for effect sizes of Social Cognition domain**

**eFigure 35. Funnel plot for effect sizes of Quality of Life domain**

**eTable 34. Results from the three-level Egger regression test.**

|  | *k* | B | SE | Z | *p* |
| --- | --- | --- | --- | --- | --- |
| **Neurocognition** | 321 | 0.064 | 0.370 | 0.172 | .863 |
| **Symptomatology** | 117 | 0.402 | 0.643 | 0.626 | .532 |
| **Functioning** | 98 | 2.673 | 0.835 | 3.200 | .001 |
| **Social cognition** | 44 | 0.388 | 1.450 | 0.268 | .789 |
| **Quality of life** | 38 | 1.083 | 0.855 | 1.266 | .206 |

*Notes*. *k* = number of effect sizes; B= unstandardized effect of the “Standard error” variable; SE= standard error.

**eReferences.**

1. Higgins, J. P., Thomas, J., Chandler, J., Cumpston, M., Li, T., Page, M. J., & Welch, V. A. (2019). *Cochrane handbook for systematic reviews of interventions.* John Wiley & Sons.
2. Bucci, S., Barrowclough, C., Ainsworth, J., Machin, M., Morris, R., Berry, K.,… Haddock, G. (2018). Actissist: Proof-of-concept trial of a theory-driven digital intervention for psychosis. *Schizophrenia Bulletin, 44*(5), 1070-1080. doi:10.1093/schbul/sby032.
3. Holzer, L., Urben, S., Passini, C. M., Jaugey, L., Herzog, M. H., Halfon, O., & Pihet, S. (2014). A randomized controlled trial of the effectiveness of computer-assisted cognitive remediation (CACR) in adolescents with psychosis or at high risk of psychosis. *Behavioural and Cognitive Psychotherapy, 42*(4), 421-434.
4. Østergaard Christensen, T., Vesterager, L., Krarup, G., Olsen, B. B., Melau, M., Gluud, C., & Nordentoft, M. (2014). Cognitive remediation combined with an early intervention service in first episode psychosis. Acta Psychiatrica Scandinavica, 130(4), 300-310.
